# Supplementary material for: Implementation of McMurchie–Davidson Algorithm for Gaussian AO Integrals Suited for SIMD Processors
Source: J Phys Chem A. 2025 Oct 13;129(42):9788–97. doi: 10.1021/acs.jpca.5c04136 (PMC12557360; doi:10.1021/acs.jpca.5c04136)
Supplement: Supplementary file 1 [file jp5c04136_si_001.pdf]

Implementation of McMurchie-Davidson algorithm for Gaussian AO integrals suited for SIMD processors.

Andrey Asadchev and Edward F. Valeev*

Department of Chemistry, Virginia Tech, Blacksburg, VA 24061

E-mail: efv@vt.edu

md2/avx0

```
# 2-center performance test
simd: OFF
cxx: 12.3.0
---
Operator: 2
K: [1,1]
results:
- (0|0):  Dims=500x500 Mem(GB)=0.00 Int/s=3.03e+06 T(MD)=0.082545 T(Ref)=1.144232 T(Ref/MD)=13.86
- (1|0):  Dims=166x500 Mem(GB)=0.00 Int/s=2.01e+06 T(MD)=0.041378 T(Ref)=0.489005 T(Ref/MD)=11.82
- (2|0):  Dims=100x500 Mem(GB)=0.00 Int/s=8.99e+05 T(MD)=0.055596 T(Ref)=0.319297 T(Ref/MD)=5.74
- (3|0):  Dims=71x500 Mem(GB)=0.00 Int/s=4.01e+05 T(MD)=0.088546 T(Ref)=0.257282 T(Ref/MD)=2.91
- (4|0):  Dims=55x500 Mem(GB)=0.00 Int/s=2.98e+05 T(MD)=0.092269 T(Ref)=0.325120 T(Ref/MD)=3.52
- (5|0):  Dims=45x500 Mem(GB)=0.00 Int/s=1.89e+05 T(MD)=0.119048 T(Ref)=0.356461 T(Ref/MD)=2.99
- (6|0):  Dims=38x500 Mem(GB)=0.00 Int/s=1.20e+05 T(MD)=0.158426 T(Ref)=0.439374 T(Ref/MD)=2.77
- (1|1):  Dims=166x166 Mem(GB)=0.00 Int/s=9.34e+05 T(MD)=0.029507 T(Ref)=0.188839 T(Ref/MD)=6.40
- (2|1):  Dims=100x166 Mem(GB)=0.00 Int/s=4.21e+05 T(MD)=0.039465 T(Ref)=0.137196 T(Ref/MD)=3.48
- (3|1):  Dims=71x166 Mem(GB)=0.00 Int/s=2.82e+05 T(MD)=0.041828 T(Ref)=0.117740 T(Ref/MD)=2.81
- (4|1):  Dims=55x166 Mem(GB)=0.00 Int/s=2.07e+05 T(MD)=0.044034 T(Ref)=0.196377 T(Ref/MD)=4.46
- (5|1):  Dims=45x166 Mem(GB)=0.00 Int/s=1.39e+05 T(MD)=0.053933 T(Ref)=0.234000 T(Ref/MD)=4.34
- (6|1):  Dims=38x166 Mem(GB)=0.00 Int/s=8.65e+04 T(MD)=0.072964 T(Ref)=0.269344 T(Ref/MD)=3.69
- (2|2):  Dims=100x100 Mem(GB)=0.00 Int/s=2.86e+05 T(MD)=0.035024 T(Ref)=0.110904 T(Ref/MD)=3.17
- (4|2):  Dims=55x100 Mem(GB)=0.00 Int/s=1.24e+05 T(MD)=0.044190 T(Ref)=0.248380 T(Ref/MD)=5.62
- (6|2):  Dims=38x100 Mem(GB)=0.00 Int/s=5.31e+04 T(MD)=0.071592 T(Ref)=0.331230 T(Ref/MD)=4.63
- (3|3):  Dims=71x71 Mem(GB)=0.00 Int/s=1.23e+05 T(MD)=0.041126 T(Ref)=0.131242 T(Ref/MD)=3.19
- (6|3):  Dims=38x71 Mem(GB)=0.00 Int/s=3.21e+04 T(MD)=0.084003 T(Ref)=0.461566 T(Ref/MD)=5.49
- (4|4):  Dims=55x55 Mem(GB)=0.00 Int/s=4.20e+04 T(MD)=0.072108 T(Ref)=0.533262 T(Ref/MD)=7.40
- (5|5):  Dims=45x45 Mem(GB)=0.00 Int/s=2.21e+04 T(MD)=0.091789 T(Ref)=0.963019 T(Ref/MD)=10.49
- (6|6):  Dims=38x38 Mem(GB)=0.00 Int/s=1.03e+04 T(MD)=0.139920 T(Ref)=1.816450 T(Ref/MD)=12.98
```

Operator: 2

K: [1,5]

results:

- (0|0): Dims=500x500 Mem(GB)=0.00 Int/s=7.40e+05 T(MD)=0.337925 T(Ref)=4.708538 T(Ref/MD)=13.93
- (1|0): Dims=166x500 Mem(GB)=0.00 Int/s=4.14e+05 T(MD)=0.200405 T(Ref)=1.854438 T(Ref/MD)=9.25
- (2|0): Dims=100x500 Mem(GB)=0.00 Int/s=1.92e+05 T(MD)=0.260441 T(Ref)=1.168532 T(Ref/MD)=4.49
- (3|0): Dims=71x500 Mem(GB)=0.00 Int/s=8.05e+04 T(MD)=0.441077 T(Ref)=0.952663 T(Ref/MD)=2.16
- (4|0): Dims=55x500 Mem(GB)=0.00 Int/s=5.99e+04 T(MD)=0.458773 T(Ref)=1.334765 T(Ref/MD)=2.91
- (5|0): Dims=45x500 Mem(GB)=0.00 Int/s=3.72e+04 T(MD)=0.604859 T(Ref)=1.624534 T(Ref/MD)=2.69
- (6|0): Dims=38x500 Mem(GB)=0.00 Int/s=2.43e+04 T(MD)=0.782130 T(Ref)=1.992155 T(Ref/MD)=2.55
- (1|1): Dims=166x166 Mem(GB)=0.00 Int/s=1.99e+05 T(MD)=0.138585 T(Ref)=0.670430 T(Ref/MD)=4.84
- (2|1): Dims=100x166 Mem(GB)=0.00 Int/s=8.72e+04 T(MD)=0.190337 T(Ref)=0.480471 T(Ref/MD)=2.52
- (3|1): Dims=71x166 Mem(GB)=0.00 Int/s=5.98e+04 T(MD)=0.197116 T(Ref)=0.418279 T(Ref/MD)=2.12
- (4|1): Dims=55x166 Mem(GB)=0.00 Int/s=4.18e+04 T(MD)=0.218351 T(Ref)=0.724604 T(Ref/MD)=3.32
- (5|1): Dims=45x166 Mem(GB)=0.00 Int/s=2.79e+04 T(MD)=0.267798 T(Ref)=0.882157 T(Ref/MD)=3.29
- (6|1): Dims=38x166 Mem(GB)=0.00 Int/s=1.74e+04 T(MD)=0.362115 T(Ref)=1.048123 T(Ref/MD)=2.89
- (2|2): Dims=100x100 Mem(GB)=0.00 Int/s=5.88e+04 T(MD)=0.169967 T(Ref)=0.375895 T(Ref/MD)=2.21
- (4|2): Dims=55x100 Mem(GB)=0.00 Int/s=2.54e+04 T(MD)=0.216891 T(Ref)=0.717268 T(Ref/MD)=3.31
- (6|2): Dims=38x100 Mem(GB)=0.00 Int/s=1.05e+04 T(MD)=0.363257 T(Ref)=1.016511 T(Ref/MD)=2.80
- (3|3): Dims=71x71 Mem(GB)=0.00 Int/s=2.50e+04 T(MD)=0.201418 T(Ref)=0.409230 T(Ref/MD)=2.03
- (6|3): Dims=38x71 Mem(GB)=0.00 Int/s=6.58e+03 T(MD)=0.409767 T(Ref)=1.170453 T(Ref/MD)=2.86
- (4|4): Dims=55x55 Mem(GB)=0.00 Int/s=8.61e+03 T(MD)=0.351424 T(Ref)=1.094576 T(Ref/MD)=3.11
- (5|5): Dims=45x45 Mem(GB)=0.00 Int/s=4.54e+03 T(MD)=0.445983 T(Ref)=1.723977 T(Ref/MD)=3.87
- (6|6): Dims=38x38 Mem(GB)=0.00 Int/s=2.13e+03 T(MD)=0.676870 T(Ref)=2.843206 T(Ref/MD)=4.20

---

md2/avx256

2-center performance test

simd: AVX 256-bits

```
cxx: Ubuntu Clang 15.0.7
---
Operator: 2
K: [1,1]
results:
- (0|0):  Dims=500x500 Mem(GB)=0.00 Int/s=2.78e+06 T(MD)=0.090068 T(Ref)=1.128647 T(Ref/MD)=12.53
- (1|0):  Dims=166x500 Mem(GB)=0.00 Int/s=3.44e+06 T(MD)=0.024129 T(Ref)=0.476319 T(Ref/MD)=19.74
- (2|0):  Dims=100x500 Mem(GB)=0.00 Int/s=1.45e+06 T(MD)=0.034430 T(Ref)=0.314124 T(Ref/MD)=9.12
- (3|0):  Dims=71x500 Mem(GB)=0.00 Int/s=1.17e+06 T(MD)=0.030236 T(Ref)=0.252793 T(Ref/MD)=8.36
- (4|0):  Dims=55x500 Mem(GB)=0.00 Int/s=9.02e+05 T(MD)=0.030472 T(Ref)=0.323833 T(Ref/MD)=10.63
- (5|0):  Dims=45x500 Mem(GB)=0.00 Int/s=5.84e+05 T(MD)=0.038547 T(Ref)=0.359691 T(Ref/MD)=9.33
- (6|0):  Dims=38x500 Mem(GB)=0.00 Int/s=3.94e+05 T(MD)=0.048247 T(Ref)=0.435897 T(Ref/MD)=9.03
- (1|1):  Dims=166x166 Mem(GB)=0.00 Int/s=1.74e+06 T(MD)=0.015830 T(Ref)=0.186515 T(Ref/MD)=11.78
- (2|1):  Dims=100x166 Mem(GB)=0.00 Int/s=9.58e+05 T(MD)=0.017326 T(Ref)=0.130691 T(Ref/MD)=7.54
- (3|1):  Dims=71x166 Mem(GB)=0.00 Int/s=6.84e+05 T(MD)=0.017229 T(Ref)=0.119977 T(Ref/MD)=6.96
- (4|1):  Dims=55x166 Mem(GB)=0.00 Int/s=5.58e+05 T(MD)=0.016356 T(Ref)=0.203431 T(Ref/MD)=12.44
- (5|1):  Dims=45x166 Mem(GB)=0.00 Int/s=3.42e+05 T(MD)=0.021825 T(Ref)=0.232665 T(Ref/MD)=10.66
- (6|1):  Dims=38x166 Mem(GB)=0.00 Int/s=2.21e+05 T(MD)=0.028522 T(Ref)=0.273483 T(Ref/MD)=9.59
- (2|2):  Dims=100x100 Mem(GB)=0.00 Int/s=6.84e+05 T(MD)=0.014616 T(Ref)=0.105744 T(Ref/MD)=7.23
- (4|2):  Dims=55x100 Mem(GB)=0.00 Int/s=3.04e+05 T(MD)=0.018084 T(Ref)=0.249072 T(Ref/MD)=13.77
- (6|2):  Dims=38x100 Mem(GB)=0.00 Int/s=1.30e+05 T(MD)=0.029310 T(Ref)=0.324520 T(Ref/MD)=11.07
- (3|3):  Dims=71x71 Mem(GB)=0.00 Int/s=2.83e+05 T(MD)=0.017839 T(Ref)=0.131004 T(Ref/MD)=7.34
- (6|3):  Dims=38x71 Mem(GB)=0.00 Int/s=6.99e+04 T(MD)=0.038603 T(Ref)=0.456820 T(Ref/MD)=11.83
- (4|4):  Dims=55x55 Mem(GB)=0.00 Int/s=1.05e+05 T(MD)=0.028879 T(Ref)=0.526290 T(Ref/MD)=18.22
- (5|5):  Dims=45x45 Mem(GB)=0.00 Int/s=4.09e+04 T(MD)=0.049457 T(Ref)=0.983537 T(Ref/MD)=19.89
- (6|6):  Dims=38x38 Mem(GB)=0.00 Int/s=1.76e+04 T(MD)=0.081946 T(Ref)=1.771513 T(Ref/MD)=21.62
---
Operator: 2
K: [1,5]
results:
- (0|0):  Dims=500x500 Mem(GB)=0.00 Int/s=1.18e+06 T(MD)=0.212318 T(Ref)=4.876732 T(Ref/MD)=22.97
- (1|0):  Dims=166x500 Mem(GB)=0.00 Int/s=8.17e+05 T(MD)=0.101606 T(Ref)=1.824714 T(Ref/MD)=17.96
- (2|0):  Dims=100x500 Mem(GB)=0.00 Int/s=5.07e+05 T(MD)=0.098700 T(Ref)=1.205870 T(Ref/MD)=12.22
```

- (3|0): Dims=71x500 Mem(GB)=0.00 Int/s=2.92e+05 T(MD)=0.121571 T(Ref)=0.966543 T(Ref/MD)=7.95
- (4|0): Dims=55x500 Mem(GB)=0.00 Int/s=2.10e+05 T(MD)=0.131114 T(Ref)=1.366513 T(Ref/MD)=10.42
- (5|0): Dims=45x500 Mem(GB)=0.00 Int/s=1.48e+05 T(MD)=0.152526 T(Ref)=1.610137 T(Ref/MD)=10.56
- (6|0): Dims=38x500 Mem(GB)=0.00 Int/s=9.36e+04 T(MD)=0.202946 T(Ref)=2.008198 T(Ref/MD)=9.90
- (1|1): Dims=166x166 Mem(GB)=0.00 Int/s=5.37e+05 T(MD)=0.051344 T(Ref)=0.681320 T(Ref/MD)=13.27
- (2|1): Dims=100x166 Mem(GB)=0.00 Int/s=2.61e+05 T(MD)=0.063721 T(Ref)=0.480423 T(Ref/MD)=7.54
- (3|1): Dims=71x166 Mem(GB)=0.00 Int/s=1.98e+05 T(MD)=0.059676 T(Ref)=0.440412 T(Ref/MD)=7.38
- (4|1): Dims=55x166 Mem(GB)=0.00 Int/s=1.32e+05 T(MD)=0.068958 T(Ref)=0.735423 T(Ref/MD)=10.66
- (5|1): Dims=45x166 Mem(GB)=0.00 Int/s=7.47e+04 T(MD)=0.099957 T(Ref)=0.875034 T(Ref/MD)=8.75
- (6|1): Dims=38x166 Mem(GB)=0.00 Int/s=4.98e+04 T(MD)=0.126742 T(Ref)=1.064559 T(Ref/MD)=8.40
- (2|2): Dims=100x100 Mem(GB)=0.00 Int/s=1.81e+05 T(MD)=0.055304 T(Ref)=0.381666 T(Ref/MD)=6.90
- (4|2): Dims=55x100 Mem(GB)=0.00 Int/s=6.71e+04 T(MD)=0.081985 T(Ref)=0.718330 T(Ref/MD)=8.76
- (6|2): Dims=38x100 Mem(GB)=0.00 Int/s=2.68e+04 T(MD)=0.141560 T(Ref)=1.006230 T(Ref/MD)=7.11
- (3|3): Dims=71x71 Mem(GB)=0.00 Int/s=6.38e+04 T(MD)=0.079057 T(Ref)=0.414717 T(Ref/MD)=5.25
- (6|3): Dims=38x71 Mem(GB)=0.00 Int/s=1.50e+04 T(MD)=0.180020 T(Ref)=1.157030 T(Ref/MD)=6.43
- (4|4): Dims=55x55 Mem(GB)=0.00 Int/s=2.32e+04 T(MD)=0.130597 T(Ref)=1.085428 T(Ref/MD)=8.31
- (5|5): Dims=45x45 Mem(GB)=0.00 Int/s=8.75e+03 T(MD)=0.231406 T(Ref)=1.744354 T(Ref/MD)=7.54
- (6|6): Dims=38x38 Mem(GB)=0.00 Int/s=3.65e+03 T(MD)=0.395314 T(Ref)=2.767897 T(Ref/MD)=7.00

---

md2/avx512

```
# 2-center performance test
simd: AVX512 512-bits
cxx: 14.2.0
---
Operator: 2
K: [1,1]
results:
- (0|0): Dims=500x500 Mem(GB)=0.00 Int/s=2.93e+06 T(MD)=0.085191 T(Ref)=1.345317 T(Ref/MD)=15.79
- (1|0): Dims=166x500 Mem(GB)=0.00 Int/s=2.62e+06 T(MD)=0.031722 T(Ref)=0.562949 T(Ref/MD)=17.75
```

- (2|0): Dims=100x500 Mem(GB)=0.00 Int/s=1.44e+06 T(MD)=0.034729 T(Ref)=0.361633 T(Ref/MD)=10.41
- (3|0): Dims=71x500 Mem(GB)=0.00 Int/s=1.12e+06 T(MD)=0.031671 T(Ref)=0.303074 T(Ref/MD)=9.57
- (4|0): Dims=55x500 Mem(GB)=0.00 Int/s=8.55e+05 T(MD)=0.032174 T(Ref)=0.364835 T(Ref/MD)=11.34
- (5|0): Dims=45x500 Mem(GB)=0.00 Int/s=5.95e+05 T(MD)=0.037796 T(Ref)=0.402405 T(Ref/MD)=10.65
- (6|0): Dims=38x500 Mem(GB)=0.00 Int/s=4.33e+05 T(MD)=0.043865 T(Ref)=0.479901 T(Ref/MD)=10.94
- (1|1): Dims=166x166 Mem(GB)=0.00 Int/s=1.54e+06 T(MD)=0.017937 T(Ref)=0.222819 T(Ref/MD)=12.42
- (2|1): Dims=100x166 Mem(GB)=0.00 Int/s=9.52e+05 T(MD)=0.017433 T(Ref)=0.162210 T(Ref/MD)=9.30
- (3|1): Dims=71x166 Mem(GB)=0.00 Int/s=6.86e+05 T(MD)=0.017184 T(Ref)=0.142899 T(Ref/MD)=8.32
- (4|1): Dims=55x166 Mem(GB)=0.00 Int/s=5.81e+05 T(MD)=0.015712 T(Ref)=0.214867 T(Ref/MD)=13.68
- (5|1): Dims=45x166 Mem(GB)=0.00 Int/s=4.08e+05 T(MD)=0.018322 T(Ref)=0.251962 T(Ref/MD)=13.75
- (6|1): Dims=38x166 Mem(GB)=0.00 Int/s=2.72e+05 T(MD)=0.023169 T(Ref)=0.304564 T(Ref/MD)=13.15
- (2|2): Dims=100x100 Mem(GB)=0.00 Int/s=6.77e+05 T(MD)=0.014777 T(Ref)=0.124854 T(Ref/MD)=8.45
- (4|2): Dims=55x100 Mem(GB)=0.00 Int/s=3.74e+05 T(MD)=0.014686 T(Ref)=0.294468 T(Ref/MD)=20.05
- (6|2): Dims=38x100 Mem(GB)=0.00 Int/s=1.71e+05 T(MD)=0.022207 T(Ref)=0.369894 T(Ref/MD)=16.66
- (3|3): Dims=71x71 Mem(GB)=0.00 Int/s=3.44e+05 T(MD)=0.014674 T(Ref)=0.139668 T(Ref/MD)=9.52
- (6|3): Dims=38x71 Mem(GB)=0.00 Int/s=1.00e+05 T(MD)=0.026884 T(Ref)=0.511711 T(Ref/MD)=19.03
- (4|4): Dims=55x55 Mem(GB)=0.00 Int/s=1.41e+05 T(MD)=0.021513 T(Ref)=0.601503 T(Ref/MD)=27.96
- (5|5): Dims=45x45 Mem(GB)=0.00 Int/s=6.66e+04 T(MD)=0.030423 T(Ref)=1.003876 T(Ref/MD)=33.00
- (6|6): Dims=38x38 Mem(GB)=0.00 Int/s=2.50e+04 T(MD)=0.057821 T(Ref)=1.657771 T(Ref/MD)=28.67

Operator: 2

K: [1,5]

results:

- (0|0): Dims=500x500 Mem(GB)=0.00 Int/s=9.29e+05 T(MD)=0.269015 T(Ref)=5.951023 T(Ref/MD)=22.12
- (1|0): Dims=166x500 Mem(GB)=0.00 Int/s=6.38e+05 T(MD)=0.130131 T(Ref)=2.244030 T(Ref/MD)=17.24
- (2|0): Dims=100x500 Mem(GB)=0.00 Int/s=4.91e+05 T(MD)=0.101924 T(Ref)=1.443165 T(Ref/MD)=14.16
- (3|0): Dims=71x500 Mem(GB)=0.00 Int/s=3.07e+05 T(MD)=0.115518 T(Ref)=1.191956 T(Ref/MD)=10.32
- (4|0): Dims=55x500 Mem(GB)=0.00 Int/s=2.24e+05 T(MD)=0.122984 T(Ref)=1.558172 T(Ref/MD)=12.67
- (5|0): Dims=45x500 Mem(GB)=0.00 Int/s=1.76e+05 T(MD)=0.127796 T(Ref)=1.875186 T(Ref/MD)=14.67
- (6|0): Dims=38x500 Mem(GB)=0.00 Int/s=1.19e+05 T(MD)=0.159307 T(Ref)=2.309512 T(Ref/MD)=14.50
- (1|1): Dims=166x166 Mem(GB)=0.00 Int/s=5.27e+05 T(MD)=0.052309 T(Ref)=0.821013 T(Ref/MD)=15.70
- (2|1): Dims=100x166 Mem(GB)=0.00 Int/s=2.78e+05 T(MD)=0.059710 T(Ref)=0.596192 T(Ref/MD)=9.98
- (3|1): Dims=71x166 Mem(GB)=0.00 Int/s=2.22e+05 T(MD)=0.053141 T(Ref)=0.520007 T(Ref/MD)=9.79

```
- (4|1):  Dims=55x166 Mem(GB)=0.00 Int/s=1.56e+05 T(MD)=0.058478 T(Ref)=0.831521 T(Ref/MD)=14.22
- (5|1):  Dims=45x166 Mem(GB)=0.00 Int/s=9.80e+04 T(MD)=0.076233 T(Ref)=0.984207 T(Ref/MD)=12.91
- (6|1):  Dims=38x166 Mem(GB)=0.00 Int/s=7.10e+04 T(MD)=0.088854 T(Ref)=1.187084 T(Ref/MD)=13.36
- (2|2):  Dims=100x100 Mem(GB)=0.00 Int/s=1.98e+05 T(MD)=0.050469 T(Ref)=0.455678 T(Ref/MD)=9.03
- (4|2):  Dims=55x100 Mem(GB)=0.00 Int/s=9.08e+04 T(MD)=0.060565 T(Ref)=0.834634 T(Ref/MD)=13.78
- (6|2):  Dims=38x100 Mem(GB)=0.00 Int/s=3.77e+04 T(MD)=0.100879 T(Ref)=1.157017 T(Ref/MD)=11.47
- (3|3):  Dims=71x71 Mem(GB)=0.00 Int/s=8.88e+04 T(MD)=0.056794 T(Ref)=0.472455 T(Ref/MD)=8.32
- (6|3):  Dims=38x71 Mem(GB)=0.00 Int/s=2.27e+04 T(MD)=0.118956 T(Ref)=1.295542 T(Ref/MD)=10.89
- (4|4):  Dims=55x55 Mem(GB)=0.00 Int/s=3.46e+04 T(MD)=0.087303 T(Ref)=1.226422 T(Ref/MD)=14.05
- (5|5):  Dims=45x45 Mem(GB)=0.00 Int/s=1.45e+04 T(MD)=0.139401 T(Ref)=1.838191 T(Ref/MD)=13.19
- (6|6):  Dims=38x38 Mem(GB)=0.00 Int/s=5.46e+03 T(MD)=0.264296 T(Ref)=2.762751 T(Ref/MD)=10.45
```

---

md2/neon

```
# 2-center performance test
simd: NEON 128-bits
cxx: Homebrew Clang 17.0.6
---
Operator: 2
K: [1,1]
results:
- (0|0):  Dims=500x500 Mem(GB)=0.00 Int/s=4.70e+06 T(MD)=0.053182 T(Ref)=0.541196 T(Ref/MD)=10.18
- (1|0):  Dims=166x500 Mem(GB)=0.00 Int/s=4.55e+06 T(MD)=0.018230 T(Ref)=0.250429 T(Ref/MD)=13.74
- (2|0):  Dims=100x500 Mem(GB)=0.00 Int/s=2.12e+06 T(MD)=0.023611 T(Ref)=0.169471 T(Ref/MD)=7.18
- (3|0):  Dims=71x500 Mem(GB)=0.00 Int/s=1.39e+06 T(MD)=0.025476 T(Ref)=0.139762 T(Ref/MD)=5.49
- (4|0):  Dims=55x500 Mem(GB)=0.00 Int/s=7.82e+05 T(MD)=0.035172 T(Ref)=0.229158 T(Ref/MD)=6.52
- (5|0):  Dims=45x500 Mem(GB)=0.00 Int/s=3.91e+05 T(MD)=0.057578 T(Ref)=0.269071 T(Ref/MD)=4.67
- (6|0):  Dims=38x500 Mem(GB)=0.00 Int/s=2.73e+05 T(MD)=0.069672 T(Ref)=0.332834 T(Ref/MD)=4.78
- (1|1):  Dims=166x166 Mem(GB)=0.00 Int/s=2.41e+06 T(MD)=0.011454 T(Ref)=0.109179 T(Ref/MD)=9.53
- (2|1):  Dims=100x166 Mem(GB)=0.00 Int/s=1.02e+06 T(MD)=0.016277 T(Ref)=0.097266 T(Ref/MD)=5.98
```

- (3|1): Dims=71x166 Mem(GB)=0.00 Int/s=5.84e+05 T(MD)=0.020198 T(Ref)=0.080480 T(Ref/MD)=3.98
- (4|1): Dims=55x166 Mem(GB)=0.00 Int/s=3.97e+05 T(MD)=0.022984 T(Ref)=0.142692 T(Ref/MD)=6.21
- (5|1): Dims=45x166 Mem(GB)=0.00 Int/s=2.64e+05 T(MD)=0.028273 T(Ref)=0.173530 T(Ref/MD)=6.14
- (6|1): Dims=38x166 Mem(GB)=0.00 Int/s=1.80e+05 T(MD)=0.035083 T(Ref)=0.193703 T(Ref/MD)=5.52
- (2|2): Dims=100x100 Mem(GB)=0.00 Int/s=6.13e+05 T(MD)=0.016325 T(Ref)=0.072950 T(Ref/MD)=4.47
- (4|2): Dims=55x100 Mem(GB)=0.00 Int/s=2.43e+05 T(MD)=0.022597 T(Ref)=0.173716 T(Ref/MD)=7.69
- (6|2): Dims=38x100 Mem(GB)=0.00 Int/s=1.10e+05 T(MD)=0.034505 T(Ref)=0.225203 T(Ref/MD)=6.53
- (3|3): Dims=71x71 Mem(GB)=0.00 Int/s=2.34e+05 T(MD)=0.021566 T(Ref)=0.072461 T(Ref/MD)=3.36
- (6|3): Dims=38x71 Mem(GB)=0.00 Int/s=6.33e+04 T(MD)=0.042597 T(Ref)=0.310459 T(Ref/MD)=7.29
- (4|4): Dims=55x55 Mem(GB)=0.00 Int/s=9.24e+04 T(MD)=0.032728 T(Ref)=0.352811 T(Ref/MD)=10.78
- (5|5): Dims=45x45 Mem(GB)=0.00 Int/s=3.58e+04 T(MD)=0.056491 T(Ref)=1.055258 T(Ref/MD)=18.68
- (6|6): Dims=38x38 Mem(GB)=0.00 Int/s=1.55e+04 T(MD)=0.092902 T(Ref)=2.331360 T(Ref/MD)=25.09

Operator: 2

K: [1,5]

results:

- (0|0): Dims=500x500 Mem(GB)=0.00 Int/s=1.80e+06 T(MD)=0.139044 T(Ref)=2.598063 T(Ref/MD)=18.69
- (1|0): Dims=166x500 Mem(GB)=0.00 Int/s=9.72e+05 T(MD)=0.085387 T(Ref)=0.948158 T(Ref/MD)=11.10
- (2|0): Dims=100x500 Mem(GB)=0.00 Int/s=6.46e+05 T(MD)=0.077447 T(Ref)=0.662366 T(Ref/MD)=8.55
- (3|0): Dims=71x500 Mem(GB)=0.00 Int/s=3.68e+05 T(MD)=0.096595 T(Ref)=0.563357 T(Ref/MD)=5.83
- (4|0): Dims=55x500 Mem(GB)=0.00 Int/s=1.78e+05 T(MD)=0.154157 T(Ref)=0.926460 T(Ref/MD)=6.01
- (5|0): Dims=45x500 Mem(GB)=0.00 Int/s=8.75e+04 T(MD)=0.257016 T(Ref)=1.204113 T(Ref/MD)=4.68
- (6|0): Dims=38x500 Mem(GB)=0.00 Int/s=5.89e+04 T(MD)=0.322678 T(Ref)=1.565536 T(Ref/MD)=4.85
- (1|1): Dims=166x166 Mem(GB)=0.00 Int/s=7.30e+05 T(MD)=0.037724 T(Ref)=0.380654 T(Ref/MD)=10.09
- (2|1): Dims=100x166 Mem(GB)=0.00 Int/s=3.48e+05 T(MD)=0.047762 T(Ref)=0.285854 T(Ref/MD)=5.98
- (3|1): Dims=71x166 Mem(GB)=0.00 Int/s=1.92e+05 T(MD)=0.061321 T(Ref)=0.253042 T(Ref/MD)=4.13
- (4|1): Dims=55x166 Mem(GB)=0.00 Int/s=8.80e+04 T(MD)=0.103733 T(Ref)=0.534062 T(Ref/MD)=5.15
- (5|1): Dims=45x166 Mem(GB)=0.00 Int/s=5.58e+04 T(MD)=0.133890 T(Ref)=0.670123 T(Ref/MD)=5.01
- (6|1): Dims=38x166 Mem(GB)=0.00 Int/s=3.82e+04 T(MD)=0.165134 T(Ref)=0.807055 T(Ref/MD)=4.89
- (2|2): Dims=100x100 Mem(GB)=0.00 Int/s=1.69e+05 T(MD)=0.059139 T(Ref)=0.227300 T(Ref/MD)=3.84
- (4|2): Dims=55x100 Mem(GB)=0.00 Int/s=5.07e+04 T(MD)=0.108532 T(Ref)=0.543409 T(Ref/MD)=5.01
- (6|2): Dims=38x100 Mem(GB)=0.00 Int/s=2.27e+04 T(MD)=0.167058 T(Ref)=0.772804 T(Ref/MD)=4.63
- (3|3): Dims=71x71 Mem(GB)=0.00 Int/s=5.09e+04 T(MD)=0.098974 T(Ref)=0.227850 T(Ref/MD)=2.30

- (6|3): Dims=38x71 Mem(GB)=0.00 Int/s=1.34e+04 T(MD)=0.201315 T(Ref)=0.859660 T(Ref/MD)=4.27
- (4|4): Dims=55x55 Mem(GB)=0.00 Int/s=1.97e+04 T(MD)=0.153860 T(Ref)=0.797253 T(Ref/MD)=5.18
- (5|5): Dims=45x45 Mem(GB)=0.00 Int/s=7.65e+03 T(MD)=0.264807 T(Ref)=1.600685 T(Ref/MD)=6.04
- (6|6): Dims=38x38 Mem(GB)=0.00 Int/s=3.29e+03 T(MD)=0.439160 T(Ref)=2.991915 T(Ref/MD)=6.81

---

md3/avx0

3-center performance test

simd: OFF

cxx: 12.3.0

blas: Intel MKL 2020.4

K: [1,1]

results:

- (0|00): Dims=2000x2000 Mem(GB)=0.03 Int/s=1.68e+08 T(MD)=0.023800 T(Ref)=0.147855 T(Ref/MD)=6.21
- (1|00): Dims=666x2000 Mem(GB)=0.03 Int/s=1.21e+08 T(MD)=0.010981 T(Ref)=0.178869 T(Ref/MD)=16.29
- (2|00): Dims=400x2000 Mem(GB)=0.03 Int/s=7.54e+07 T(MD)=0.010617 T(Ref)=0.120681 T(Ref/MD)=11.37
- (4|00): Dims=222x2000 Mem(GB)=0.03 Int/s=3.86e+07 T(MD)=0.011511 T(Ref)=0.089655 T(Ref/MD)=7.79
- (6|00): Dims=153x2000 Mem(GB)=0.03 Int/s=2.44e+07 T(MD)=0.012565 T(Ref)=0.086152 T(Ref/MD)=6.86
- (0|11): Dims=2000x222 Mem(GB)=0.03 Int/s=3.69e+07 T(MD)=0.012030 T(Ref)=0.068559 T(Ref/MD)=5.70
- (1|11): Dims=666x222 Mem(GB)=0.03 Int/s=1.96e+07 T(MD)=0.007561 T(Ref)=0.029394 T(Ref/MD)=3.89
- (2|11): Dims=400x222 Mem(GB)=0.03 Int/s=1.19e+07 T(MD)=0.007433 T(Ref)=0.028767 T(Ref/MD)=3.87
- (4|11): Dims=222x222 Mem(GB)=0.03 Int/s=5.01e+06 T(MD)=0.009847 T(Ref)=0.026891 T(Ref/MD)=2.73
- (6|11): Dims=153x222 Mem(GB)=0.03 Int/s=2.52e+06 T(MD)=0.013504 T(Ref)=0.037727 T(Ref/MD)=2.79
- (1|22): Dims=666x80 Mem(GB)=0.03 Int/s=5.35e+06 T(MD)=0.009955 T(Ref)=0.028014 T(Ref/MD)=2.81
- (2|22): Dims=400x80 Mem(GB)=0.03 Int/s=3.40e+06 T(MD)=0.009407 T(Ref)=0.027591 T(Ref/MD)=2.93
- (1|33): Dims=666x40 Mem(GB)=0.03 Int/s=1.39e+06 T(MD)=0.019136 T(Ref)=0.038532 T(Ref/MD)=2.01
- (3|33): Dims=285x40 Mem(GB)=0.03 Int/s=5.30e+05 T(MD)=0.021518 T(Ref)=0.047911 T(Ref/MD)=2.23
- (1|44): Dims=666x24 Mem(GB)=0.03 Int/s=4.10e+05 T(MD)=0.038942 T(Ref)=0.062901 T(Ref/MD)=1.62
- (4|44): Dims=222x24 Mem(GB)=0.03 Int/s=1.27e+05 T(MD)=0.041804 T(Ref)=0.113687 T(Ref/MD)=2.72

```
- (1|55): Dims=666x16 Mem(GB)=0.03 Int/s=1.30e+05 T(MD)=0.081669 T(Ref)=0.097548 T(Ref/MD)=1.19
- (5|55): Dims=181x16 Mem(GB)=0.03 Int/s=3.04e+04 T(MD)=0.095397 T(Ref)=0.229492 T(Ref/MD)=2.41
- (1|66): Dims=666x11 Mem(GB)=0.03 Int/s=3.48e+04 T(MD)=0.210671 T(Ref)=0.151206 T(Ref/MD)=0.72
- (6|66): Dims=153x11 Mem(GB)=0.03 Int/s=1.24e+04 T(MD)=0.135348 T(Ref)=0.387052 T(Ref/MD)=2.86
---
K: [1,10]
results:
- (0|00): Dims=2000x2000 Mem(GB)=0.03 Int/s=1.85e+07 T(MD)=0.216570 T(Ref)=0.972695 T(Ref/MD)=4.49
- (1|00): Dims=666x2000 Mem(GB)=0.03 Int/s=1.29e+07 T(MD)=0.103028 T(Ref)=0.602893 T(Ref/MD)=5.85
- (2|00): Dims=400x2000 Mem(GB)=0.03 Int/s=8.02e+06 T(MD)=0.099737 T(Ref)=0.404369 T(Ref/MD)=4.05
- (4|00): Dims=222x2000 Mem(GB)=0.03 Int/s=4.02e+06 T(MD)=0.110454 T(Ref)=0.301660 T(Ref/MD)=2.73
- (6|00): Dims=153x2000 Mem(GB)=0.03 Int/s=2.46e+06 T(MD)=0.124539 T(Ref)=0.285962 T(Ref/MD)=2.30
- (0|11): Dims=2000x222 Mem(GB)=0.03 Int/s=3.84e+06 T(MD)=0.115588 T(Ref)=0.231800 T(Ref/MD)=2.01
- (1|11): Dims=666x222 Mem(GB)=0.03 Int/s=1.99e+06 T(MD)=0.074213 T(Ref)=0.093378 T(Ref/MD)=1.26
- (2|11): Dims=400x222 Mem(GB)=0.03 Int/s=1.18e+06 T(MD)=0.075052 T(Ref)=0.078369 T(Ref/MD)=1.04
- (4|11): Dims=222x222 Mem(GB)=0.03 Int/s=4.87e+05 T(MD)=0.101266 T(Ref)=0.113199 T(Ref/MD)=1.12
- (6|11): Dims=153x222 Mem(GB)=0.03 Int/s=2.45e+05 T(MD)=0.138606 T(Ref)=0.198742 T(Ref/MD)=1.43
- (1|22): Dims=666x80 Mem(GB)=0.03 Int/s=5.44e+05 T(MD)=0.097864 T(Ref)=0.096496 T(Ref/MD)=0.99
- (2|22): Dims=400x80 Mem(GB)=0.03 Int/s=3.31e+05 T(MD)=0.096794 T(Ref)=0.097268 T(Ref/MD)=1.00
- (1|33): Dims=666x40 Mem(GB)=0.03 Int/s=1.05e+05 T(MD)=0.253143 T(Ref)=0.120251 T(Ref/MD)=0.48
- (3|33): Dims=285x40 Mem(GB)=0.03 Int/s=4.56e+04 T(MD)=0.250064 T(Ref)=0.164063 T(Ref/MD)=0.66
- (1|44): Dims=666x24 Mem(GB)=0.03 Int/s=3.08e+04 T(MD)=0.519292 T(Ref)=0.177015 T(Ref/MD)=0.34
- (4|44): Dims=222x24 Mem(GB)=0.03 Int/s=1.10e+04 T(MD)=0.482723 T(Ref)=0.521547 T(Ref/MD)=1.08
- (1|55): Dims=666x16 Mem(GB)=0.03 Int/s=1.05e+04 T(MD)=1.014872 T(Ref)=0.253817 T(Ref/MD)=0.25
- (5|55): Dims=181x16 Mem(GB)=0.03 Int/s=2.92e+03 T(MD)=0.990982 T(Ref)=1.015953 T(Ref/MD)=1.03
- (1|66): Dims=666x11 Mem(GB)=0.03 Int/s=3.44e+03 T(MD)=2.132248 T(Ref)=0.339673 T(Ref/MD)=0.16
- (6|66): Dims=153x11 Mem(GB)=0.03 Int/s=1.19e+03 T(MD)=1.411778 T(Ref)=1.662849 T(Ref/MD)=1.18
---
K: [5,10]
results:
- (0|00): Dims=2000x2000 Mem(GB)=0.03 Int/s=3.57e+06 T(MD)=1.120345 T(Ref)=4.599164 T(Ref/MD)=4.11
- (1|00): Dims=666x2000 Mem(GB)=0.03 Int/s=2.57e+06 T(MD)=0.518898 T(Ref)=2.558848 T(Ref/MD)=4.93
- (2|00): Dims=400x2000 Mem(GB)=0.03 Int/s=1.61e+06 T(MD)=0.496860 T(Ref)=1.640197 T(Ref/MD)=3.30
```

- (4|00): Dims=222x2000 Mem(GB)=0.03 Int/s=8.01e+05 T(MD)=0.554141 T(Ref)=1.254637 T(Ref/MD)=2.26
- (6|00): Dims=153x2000 Mem(GB)=0.03 Int/s=4.81e+05 T(MD)=0.636753 T(Ref)=1.153944 T(Ref/MD)=1.81
- (0|11): Dims=2000x222 Mem(GB)=0.03 Int/s=7.64e+05 T(MD)=0.581125 T(Ref)=0.969398 T(Ref/MD)=1.67
- (1|11): Dims=666x222 Mem(GB)=0.03 Int/s=3.99e+05 T(MD)=0.370912 T(Ref)=0.378794 T(Ref/MD)=1.02
- (2|11): Dims=400x222 Mem(GB)=0.03 Int/s=2.34e+05 T(MD)=0.379148 T(Ref)=0.300668 T(Ref/MD)=0.79
- (4|11): Dims=222x222 Mem(GB)=0.03 Int/s=9.71e+04 T(MD)=0.507777 T(Ref)=0.494557 T(Ref/MD)=0.97
- (6|11): Dims=153x222 Mem(GB)=0.03 Int/s=4.86e+04 T(MD)=0.698817 T(Ref)=0.932568 T(Ref/MD)=1.33
- (1|22): Dims=666x80 Mem(GB)=0.03 Int/s=4.26e+05 T(MD)=0.125038 T(Ref)=0.400124 T(Ref/MD)=3.20
- (2|22): Dims=400x80 Mem(GB)=0.03 Int/s=2.82e+05 T(MD)=0.113318 T(Ref)=0.405542 T(Ref/MD)=3.58
- (1|33): Dims=666x40 Mem(GB)=0.03 Int/s=9.51e+04 T(MD)=0.280101 T(Ref)=0.483734 T(Ref/MD)=1.73
- (3|33): Dims=285x40 Mem(GB)=0.03 Int/s=4.40e+04 T(MD)=0.259329 T(Ref)=0.681173 T(Ref/MD)=2.63
- (1|44): Dims=666x24 Mem(GB)=0.03 Int/s=2.99e+04 T(MD)=0.533868 T(Ref)=0.685992 T(Ref/MD)=1.28
- (4|44): Dims=222x24 Mem(GB)=0.03 Int/s=1.09e+04 T(MD)=0.489490 T(Ref)=2.330436 T(Ref/MD)=4.76
- (1|55): Dims=666x16 Mem(GB)=0.03 Int/s=1.04e+04 T(MD)=1.025700 T(Ref)=0.932790 T(Ref/MD)=0.91
- (5|55): Dims=181x16 Mem(GB)=0.03 Int/s=2.90e+03 T(MD)=0.997729 T(Ref)=4.556620 T(Ref/MD)=4.57
- (1|66): Dims=666x11 Mem(GB)=0.03 Int/s=3.41e+03 T(MD)=2.148722 T(Ref)=1.175561 T(Ref/MD)=0.55
- (6|66): Dims=153x11 Mem(GB)=0.03 Int/s=1.19e+03 T(MD)=1.415478 T(Ref)=7.380411 T(Ref/MD)=5.21

---

md3/avx256

```
# 3-center performance test
simd: AVX 256-bits
cxx: Ubuntu Clang 15.0.7
blas: Intel MKL 2020.4
---
K: [1,1]
results:
- (0|00): Dims=2000x2000 Mem(GB)=0.03 Int/s=1.87e+08 T(MD)=0.021430 T(Ref)=0.179589 T(Ref/MD)=8.38
- (1|00): Dims=666x2000 Mem(GB)=0.03 Int/s=1.68e+08 T(MD)=0.007916 T(Ref)=0.236395 T(Ref/MD)=29.86
- (2|00): Dims=400x2000 Mem(GB)=0.03 Int/s=1.34e+08 T(MD)=0.005956 T(Ref)=0.163047 T(Ref/MD)=27.38
```

- (4|00): Dims=222x2000 Mem(GB)=0.03 Int/s=8.13e+07 T(MD)=0.005462 T(Ref)=0.130342 T(Ref/MD)=23.86

- (6|00): Dims=153x2000 Mem(GB)=0.03 Int/s=5.81e+07 T(MD)=0.005266 T(Ref)=0.135546 T(Ref/MD)=25.74

- (0|11): Dims=2000x222 Mem(GB)=0.03 Int/s=8.81e+07 T(MD)=0.005042 T(Ref)=0.089728 T(Ref/MD)=17.80

- (1|11): Dims=666x222 Mem(GB)=0.03 Int/s=5.09e+07 T(MD)=0.002907 T(Ref)=0.039194 T(Ref/MD)=13.48

- (2|11): Dims=400x222 Mem(GB)=0.03 Int/s=2.85e+07 T(MD)=0.003118 T(Ref)=0.032362 T(Ref/MD)=10.38

- (4|11): Dims=222x222 Mem(GB)=0.03 Int/s=1.36e+07 T(MD)=0.003633 T(Ref)=0.035586 T(Ref/MD)=9.80

- (6|11): Dims=153x222 Mem(GB)=0.03 Int/s=7.59e+06 T(MD)=0.004475 T(Ref)=0.049646 T(Ref/MD)=11.09

- (1|22): Dims=666x80 Mem(GB)=0.03 Int/s=8.00e+06 T(MD)=0.006658 T(Ref)=0.034779 T(Ref/MD)=5.22

- (2|22): Dims=400x80 Mem(GB)=0.03 Int/s=5.25e+06 T(MD)=0.006094 T(Ref)=0.032994 T(Ref/MD)=5.41

- (1|33): Dims=666x40 Mem(GB)=0.03 Int/s=1.88e+06 T(MD)=0.014159 T(Ref)=0.045241 T(Ref/MD)=3.20

- (3|33): Dims=285x40 Mem(GB)=0.03 Int/s=7.14e+05 T(MD)=0.015976 T(Ref)=0.054641 T(Ref/MD)=3.42

- (1|44): Dims=666x24 Mem(GB)=0.03 Int/s=4.65e+05 T(MD)=0.034401 T(Ref)=0.069854 T(Ref/MD)=2.03

- (4|44): Dims=222x24 Mem(GB)=0.03 Int/s=1.59e+05 T(MD)=0.033573 T(Ref)=0.127045 T(Ref/MD)=3.78

- (1|55): Dims=666x16 Mem(GB)=0.03 Int/s=1.71e+05 T(MD)=0.062486 T(Ref)=0.106819 T(Ref/MD)=1.71

- (5|55): Dims=181x16 Mem(GB)=0.03 Int/s=4.60e+04 T(MD)=0.062895 T(Ref)=0.242251 T(Ref/MD)=3.85

- (1|66): Dims=666x11 Mem(GB)=0.03 Int/s=7.22e+04 T(MD)=0.101524 T(Ref)=0.160788 T(Ref/MD)=1.58

- (6|66): Dims=153x11 Mem(GB)=0.03 Int/s=1.78e+04 T(MD)=0.094712 T(Ref)=0.414039 T(Ref/MD)=4.37

K: [1,10]

results:

- (0|00): Dims=2000x2000 Mem(GB)=0.03 Int/s=2.02e+07 T(MD)=0.198324 T(Ref)=1.098841 T(Ref/MD)=5.54

- (1|00): Dims=666x2000 Mem(GB)=0.03 Int/s=1.92e+07 T(MD)=0.069343 T(Ref)=0.679947 T(Ref/MD)=9.81

- (2|00): Dims=400x2000 Mem(GB)=0.03 Int/s=1.59e+07 T(MD)=0.050315 T(Ref)=0.445753 T(Ref/MD)=8.86

- (4|00): Dims=222x2000 Mem(GB)=0.03 Int/s=9.23e+06 T(MD)=0.048117 T(Ref)=0.356620 T(Ref/MD)=7.41

- (6|00): Dims=153x2000 Mem(GB)=0.03 Int/s=6.89e+06 T(MD)=0.044432 T(Ref)=0.335193 T(Ref/MD)=7.54

- (0|11): Dims=2000x222 Mem(GB)=0.03 Int/s=9.70e+06 T(MD)=0.045777 T(Ref)=0.260337 T(Ref/MD)=5.69

- (1|11): Dims=666x222 Mem(GB)=0.03 Int/s=5.90e+06 T(MD)=0.025076 T(Ref)=0.104949 T(Ref/MD)=4.19

- (2|11): Dims=400x222 Mem(GB)=0.03 Int/s=3.38e+06 T(MD)=0.026277 T(Ref)=0.085358 T(Ref/MD)=3.25

- (4|11): Dims=222x222 Mem(GB)=0.03 Int/s=1.56e+06 T(MD)=0.031625 T(Ref)=0.123953 T(Ref/MD)=3.92

- (6|11): Dims=153x222 Mem(GB)=0.03 Int/s=8.38e+05 T(MD)=0.040549 T(Ref)=0.222508 T(Ref/MD)=5.49

- (1|22): Dims=666x80 Mem(GB)=0.03 Int/s=8.54e+05 T(MD)=0.062367 T(Ref)=0.106409 T(Ref/MD)=1.71

- (2|22): Dims=400x80 Mem(GB)=0.03 Int/s=5.62e+05 T(MD)=0.056908 T(Ref)=0.103281 T(Ref/MD)=1.81

- (1|33): Dims=666x40 Mem(GB)=0.03 Int/s=1.76e+05 T(MD)=0.151534 T(Ref)=0.129358 T(Ref/MD)=0.85

```
- (3|33): Dims=285x40 Mem(GB)=0.03 Int/s=7.05e+04 T(MD)=0.161762 T(Ref)=0.172285 T(Ref/MD)=1.07
- (1|44): Dims=666x24 Mem(GB)=0.03 Int/s=4.25e+04 T(MD)=0.375948 T(Ref)=0.184392 T(Ref/MD)=0.49
- (4|44): Dims=222x24 Mem(GB)=0.03 Int/s=1.57e+04 T(MD)=0.339400 T(Ref)=0.543907 T(Ref/MD)=1.60
- (1|55): Dims=666x16 Mem(GB)=0.03 Int/s=1.69e+04 T(MD)=0.629355 T(Ref)=0.262160 T(Ref/MD)=0.42
- (5|55): Dims=181x16 Mem(GB)=0.03 Int/s=4.63e+03 T(MD)=0.626061 T(Ref)=1.055938 T(Ref/MD)=1.69
- (1|66): Dims=666x11 Mem(GB)=0.03 Int/s=7.34e+03 T(MD)=0.997827 T(Ref)=0.348205 T(Ref/MD)=0.35
- (6|66): Dims=153x11 Mem(GB)=0.03 Int/s=1.81e+03 T(MD)=0.927852 T(Ref)=1.719442 T(Ref/MD)=1.85
---
K: [5,10]
results:
- (0|00): Dims=2000x2000 Mem(GB)=0.03 Int/s=4.08e+06 T(MD)=0.980041 T(Ref)=5.140732 T(Ref/MD)=5.25
- (1|00): Dims=666x2000 Mem(GB)=0.03 Int/s=3.87e+06 T(MD)=0.343937 T(Ref)=2.642276 T(Ref/MD)=7.68
- (2|00): Dims=400x2000 Mem(GB)=0.03 Int/s=3.26e+06 T(MD)=0.245101 T(Ref)=1.694142 T(Ref/MD)=6.91
- (4|00): Dims=222x2000 Mem(GB)=0.03 Int/s=1.93e+06 T(MD)=0.229807 T(Ref)=1.331395 T(Ref/MD)=5.79
- (6|00): Dims=153x2000 Mem(GB)=0.03 Int/s=1.43e+06 T(MD)=0.214382 T(Ref)=1.201425 T(Ref/MD)=5.60
- (0|11): Dims=2000x222 Mem(GB)=0.03 Int/s=1.98e+06 T(MD)=0.224574 T(Ref)=1.007683 T(Ref/MD)=4.49
- (1|11): Dims=666x222 Mem(GB)=0.03 Int/s=1.22e+06 T(MD)=0.120776 T(Ref)=0.395219 T(Ref/MD)=3.27
- (2|11): Dims=400x222 Mem(GB)=0.03 Int/s=6.90e+05 T(MD)=0.128746 T(Ref)=0.311075 T(Ref/MD)=2.42
- (4|11): Dims=222x222 Mem(GB)=0.03 Int/s=3.19e+05 T(MD)=0.154280 T(Ref)=0.504176 T(Ref/MD)=3.27
- (6|11): Dims=153x222 Mem(GB)=0.03 Int/s=1.71e+05 T(MD)=0.198267 T(Ref)=0.980946 T(Ref/MD)=4.95
- (1|22): Dims=666x80 Mem(GB)=0.03 Int/s=6.73e+05 T(MD)=0.079157 T(Ref)=0.417283 T(Ref/MD)=5.27
- (2|22): Dims=400x80 Mem(GB)=0.03 Int/s=4.85e+05 T(MD)=0.066021 T(Ref)=0.407972 T(Ref/MD)=6.18
- (1|33): Dims=666x40 Mem(GB)=0.03 Int/s=1.63e+05 T(MD)=0.163706 T(Ref)=0.500624 T(Ref/MD)=3.06
- (3|33): Dims=285x40 Mem(GB)=0.03 Int/s=6.75e+04 T(MD)=0.168911 T(Ref)=0.689693 T(Ref/MD)=4.08
- (1|44): Dims=666x24 Mem(GB)=0.03 Int/s=4.20e+04 T(MD)=0.380508 T(Ref)=0.683499 T(Ref/MD)=1.80
- (4|44): Dims=222x24 Mem(GB)=0.03 Int/s=1.58e+04 T(MD)=0.337044 T(Ref)=2.365398 T(Ref/MD)=7.02
- (1|55): Dims=666x16 Mem(GB)=0.03 Int/s=1.70e+04 T(MD)=0.626063 T(Ref)=0.940073 T(Ref/MD)=1.50
- (5|55): Dims=181x16 Mem(GB)=0.03 Int/s=4.70e+03 T(MD)=0.616222 T(Ref)=4.555452 T(Ref/MD)=7.39
- (1|66): Dims=666x11 Mem(GB)=0.03 Int/s=7.40e+03 T(MD)=0.989531 T(Ref)=1.173745 T(Ref/MD)=1.19
- (6|66): Dims=153x11 Mem(GB)=0.03 Int/s=1.85e+03 T(MD)=0.911696 T(Ref)=7.417046 T(Ref/MD)=8.14
```

md3/avx512

```
# 3-center performance test
simd: AVX512 512-bits
cxx: 14.2.0
blas: Intel MKL 2025.1
---
K: [1,1]
results:
- (0|00): Dims=2000x2000 Mem(GB)=0.03 Int/s=1.88e+08 T(MD)=0.021261 T(Ref)=0.169684 T(Ref/MD)=7.98
- (1|00): Dims=666x2000 Mem(GB)=0.03 Int/s=1.71e+08 T(MD)=0.007808 T(Ref)=0.195276 T(Ref/MD)=25.01
- (2|00): Dims=400x2000 Mem(GB)=0.03 Int/s=1.41e+08 T(MD)=0.005688 T(Ref)=0.131744 T(Ref/MD)=23.16
- (4|00): Dims=222x2000 Mem(GB)=0.03 Int/s=9.69e+07 T(MD)=0.004581 T(Ref)=0.100434 T(Ref/MD)=21.92
- (6|00): Dims=153x2000 Mem(GB)=0.03 Int/s=7.71e+07 T(MD)=0.003969 T(Ref)=0.100007 T(Ref/MD)=25.20
- (0|11): Dims=2000x222 Mem(GB)=0.03 Int/s=8.72e+07 T(MD)=0.005092 T(Ref)=0.075800 T(Ref/MD)=14.88
- (1|11): Dims=666x222 Mem(GB)=0.03 Int/s=5.24e+07 T(MD)=0.002820 T(Ref)=0.032809 T(Ref/MD)=11.64
- (2|11): Dims=400x222 Mem(GB)=0.03 Int/s=3.86e+07 T(MD)=0.002298 T(Ref)=0.026795 T(Ref/MD)=11.66
- (4|11): Dims=222x222 Mem(GB)=0.03 Int/s=2.13e+07 T(MD)=0.002309 T(Ref)=0.032166 T(Ref/MD)=13.93
- (6|11): Dims=153x222 Mem(GB)=0.03 Int/s=1.13e+07 T(MD)=0.003009 T(Ref)=0.045646 T(Ref/MD)=15.17
- (1|22): Dims=666x80 Mem(GB)=0.03 Int/s=1.14e+07 T(MD)=0.004678 T(Ref)=0.031130 T(Ref/MD)=6.66
- (2|22): Dims=400x80 Mem(GB)=0.03 Int/s=6.83e+06 T(MD)=0.004685 T(Ref)=0.030909 T(Ref/MD)=6.60
- (1|33): Dims=666x40 Mem(GB)=0.03 Int/s=2.97e+06 T(MD)=0.008970 T(Ref)=0.043570 T(Ref/MD)=4.86
- (3|33): Dims=285x40 Mem(GB)=0.03 Int/s=9.60e+05 T(MD)=0.011871 T(Ref)=0.052770 T(Ref/MD)=4.45
- (1|44): Dims=666x24 Mem(GB)=0.03 Int/s=9.06e+05 T(MD)=0.017642 T(Ref)=0.069663 T(Ref/MD)=3.95
- (4|44): Dims=222x24 Mem(GB)=0.03 Int/s=2.73e+05 T(MD)=0.019504 T(Ref)=0.125764 T(Ref/MD)=6.45
- (1|55): Dims=666x16 Mem(GB)=0.03 Int/s=3.35e+05 T(MD)=0.031796 T(Ref)=0.104630 T(Ref/MD)=3.29
- (5|55): Dims=181x16 Mem(GB)=0.03 Int/s=8.17e+04 T(MD)=0.035434 T(Ref)=0.240364 T(Ref/MD)=6.78
- (1|66): Dims=666x11 Mem(GB)=0.03 Int/s=1.36e+05 T(MD)=0.053930 T(Ref)=0.148128 T(Ref/MD)=2.75
- (6|66): Dims=153x11 Mem(GB)=0.03 Int/s=2.59e+04 T(MD)=0.064985 T(Ref)=0.439600 T(Ref/MD)=6.76
---
K: [1,10]
results:
- (0|00): Dims=2000x2000 Mem(GB)=0.03 Int/s=2.09e+07 T(MD)=0.191623 T(Ref)=1.107581 T(Ref/MD)=5.78
```

- (1|00): Dims=666x2000 Mem(GB)=0.03 Int/s=1.99e+07 T(MD)=0.066863 T(Ref)=0.652141 T(Ref/MD)=9.75

- (2|00): Dims=400x2000 Mem(GB)=0.03 Int/s=1.73e+07 T(MD)=0.046354 T(Ref)=0.435154 T(Ref/MD)=9.39

- (4|00): Dims=222x2000 Mem(GB)=0.03 Int/s=1.25e+07 T(MD)=0.035471 T(Ref)=0.327684 T(Ref/MD)=9.24

- (6|00): Dims=153x2000 Mem(GB)=0.03 Int/s=9.99e+06 T(MD)=0.030642 T(Ref)=0.327141 T(Ref/MD)=10.68

- (0|11): Dims=2000x222 Mem(GB)=0.03 Int/s=1.04e+07 T(MD)=0.042774 T(Ref)=0.262840 T(Ref/MD)=6.14

- (1|11): Dims=666x222 Mem(GB)=0.03 Int/s=6.25e+06 T(MD)=0.023663 T(Ref)=0.103162 T(Ref/MD)=4.36

- (2|11): Dims=400x222 Mem(GB)=0.03 Int/s=4.53e+06 T(MD)=0.019612 T(Ref)=0.081874 T(Ref/MD)=4.17

- (4|11): Dims=222x222 Mem(GB)=0.03 Int/s=2.58e+06 T(MD)=0.019134 T(Ref)=0.126087 T(Ref/MD)=6.59

- (6|11): Dims=153x222 Mem(GB)=0.03 Int/s=1.33e+06 T(MD)=0.025560 T(Ref)=0.228060 T(Ref/MD)=8.92

- (1|22): Dims=666x80 Mem(GB)=0.03 Int/s=1.25e+06 T(MD)=0.042789 T(Ref)=0.106896 T(Ref/MD)=2.50

- (2|22): Dims=400x80 Mem(GB)=0.03 Int/s=7.40e+05 T(MD)=0.043265 T(Ref)=0.106068 T(Ref/MD)=2.45

- (1|33): Dims=666x40 Mem(GB)=0.03 Int/s=3.15e+05 T(MD)=0.084492 T(Ref)=0.133025 T(Ref/MD)=1.57

- (3|33): Dims=285x40 Mem(GB)=0.03 Int/s=9.53e+04 T(MD)=0.119612 T(Ref)=0.179722 T(Ref/MD)=1.50

- (1|44): Dims=666x24 Mem(GB)=0.03 Int/s=7.82e+04 T(MD)=0.204324 T(Ref)=0.194154 T(Ref/MD)=0.95

- (4|44): Dims=222x24 Mem(GB)=0.03 Int/s=2.47e+04 T(MD)=0.215597 T(Ref)=0.549251 T(Ref/MD)=2.55

- (1|55): Dims=666x16 Mem(GB)=0.03 Int/s=2.29e+04 T(MD)=0.465690 T(Ref)=0.267962 T(Ref/MD)=0.58

- (5|55): Dims=181x16 Mem(GB)=0.03 Int/s=7.49e+03 T(MD)=0.386694 T(Ref)=1.060818 T(Ref/MD)=2.74

- (1|66): Dims=666x11 Mem(GB)=0.03 Int/s=9.83e+03 T(MD)=0.745215 T(Ref)=0.351466 T(Ref/MD)=0.47

- (6|66): Dims=153x11 Mem(GB)=0.03 Int/s=2.60e+03 T(MD)=0.646163 T(Ref)=1.799980 T(Ref/MD)=2.79

K: [5,10]

results:

- (0|00): Dims=2000x2000 Mem(GB)=0.03 Int/s=4.06e+06 T(MD)=0.986054 T(Ref)=5.300746 T(Ref/MD)=5.38

- (1|00): Dims=666x2000 Mem(GB)=0.03 Int/s=3.89e+06 T(MD)=0.342061 T(Ref)=2.725663 T(Ref/MD)=7.97

- (2|00): Dims=400x2000 Mem(GB)=0.03 Int/s=3.49e+06 T(MD)=0.229182 T(Ref)=1.786971 T(Ref/MD)=7.80

- (4|00): Dims=222x2000 Mem(GB)=0.03 Int/s=2.54e+06 T(MD)=0.175092 T(Ref)=1.402284 T(Ref/MD)=8.01

- (6|00): Dims=153x2000 Mem(GB)=0.03 Int/s=2.03e+06 T(MD)=0.151103 T(Ref)=1.319594 T(Ref/MD)=8.73

- (0|11): Dims=2000x222 Mem(GB)=0.03 Int/s=2.06e+06 T(MD)=0.215111 T(Ref)=1.105420 T(Ref/MD)=5.14

- (1|11): Dims=666x222 Mem(GB)=0.03 Int/s=1.27e+06 T(MD)=0.116832 T(Ref)=0.422015 T(Ref/MD)=3.61

- (2|11): Dims=400x222 Mem(GB)=0.03 Int/s=9.21e+05 T(MD)=0.096447 T(Ref)=0.329836 T(Ref/MD)=3.42

- (4|11): Dims=222x222 Mem(GB)=0.03 Int/s=5.32e+05 T(MD)=0.092685 T(Ref)=0.540545 T(Ref/MD)=5.83

- (6|11): Dims=153x222 Mem(GB)=0.03 Int/s=2.74e+05 T(MD)=0.124088 T(Ref)=1.040427 T(Ref/MD)=8.38

- (1|22): Dims=666x80 Mem(GB)=0.03 Int/s=8.73e+05 T(MD)=0.061031 T(Ref)=0.444227 T(Ref/MD)=7.28

- (2|22): Dims=400x80 Mem(GB)=0.03 Int/s=5.82e+05 T(MD)=0.054978 T(Ref)=0.441173 T(Ref/MD)=8.02
- (1|33): Dims=666x40 Mem(GB)=0.03 Int/s=2.73e+05 T(MD)=0.097484 T(Ref)=0.535168 T(Ref/MD)=5.49
- (3|33): Dims=285x40 Mem(GB)=0.03 Int/s=9.04e+04 T(MD)=0.126153 T(Ref)=0.743412 T(Ref/MD)=5.89
- (1|44): Dims=666x24 Mem(GB)=0.03 Int/s=7.10e+04 T(MD)=0.225094 T(Ref)=0.750359 T(Ref/MD)=3.33
- (4|44): Dims=222x24 Mem(GB)=0.03 Int/s=2.42e+04 T(MD)=0.220304 T(Ref)=2.430095 T(Ref/MD)=11.03
- (1|55): Dims=666x16 Mem(GB)=0.03 Int/s=2.23e+04 T(MD)=0.478374 T(Ref)=0.992874 T(Ref/MD)=2.08
- (5|55): Dims=181x16 Mem(GB)=0.03 Int/s=7.41e+03 T(MD)=0.390755 T(Ref)=4.755806 T(Ref/MD)=12.17
- (1|66): Dims=666x11 Mem(GB)=0.03 Int/s=9.67e+03 T(MD)=0.757221 T(Ref)=1.254659 T(Ref/MD)=1.66
- (6|66): Dims=153x11 Mem(GB)=0.03 Int/s=2.59e+03 T(MD)=0.649110 T(Ref)=7.844474 T(Ref/MD)=12.08

---

md3/neon

```
# 3-center performance test
simd: NEON 128-bits
cxx: Homebrew Clang 17.0.6
blas: Apple Accelerate
---
K: [1,1]
results:
- (0|00): Dims=2000x2000 Mem(GB)=0.03 Int/s=2.54e+08 T(MD)=0.015741 T(Ref)=0.095324 T(Ref/MD)=6.06
- (1|00): Dims=666x2000 Mem(GB)=0.03 Int/s=2.09e+08 T(MD)=0.006384 T(Ref)=0.092772 T(Ref/MD)=14.53
- (2|00): Dims=400x2000 Mem(GB)=0.03 Int/s=1.52e+08 T(MD)=0.005255 T(Ref)=0.065778 T(Ref/MD)=12.52
- (4|00): Dims=222x2000 Mem(GB)=0.03 Int/s=8.98e+07 T(MD)=0.004945 T(Ref)=0.059603 T(Ref/MD)=12.05
- (6|00): Dims=153x2000 Mem(GB)=0.03 Int/s=4.83e+07 T(MD)=0.006334 T(Ref)=0.060334 T(Ref/MD)=9.53
- (0|11): Dims=2000x222 Mem(GB)=0.03 Int/s=7.74e+07 T(MD)=0.005740 T(Ref)=0.041626 T(Ref/MD)=7.25
- (1|11): Dims=666x222 Mem(GB)=0.03 Int/s=4.02e+07 T(MD)=0.003680 T(Ref)=0.019670 T(Ref/MD)=5.35
- (2|11): Dims=400x222 Mem(GB)=0.03 Int/s=2.51e+07 T(MD)=0.003541 T(Ref)=0.015002 T(Ref/MD)=4.24
- (4|11): Dims=222x222 Mem(GB)=0.03 Int/s=1.44e+07 T(MD)=0.003421 T(Ref)=0.019825 T(Ref/MD)=5.80
- (6|11): Dims=153x222 Mem(GB)=0.03 Int/s=8.31e+06 T(MD)=0.004088 T(Ref)=0.027643 T(Ref/MD)=6.76
- (1|22): Dims=666x80 Mem(GB)=0.03 Int/s=7.41e+06 T(MD)=0.007189 T(Ref)=0.021702 T(Ref/MD)=3.02
```

- (2|22): Dims=400x80 Mem(GB)=0.03 Int/s=4.67e+06 T(MD)=0.006847 T(Ref)=0.021089 T(Ref/MD)=3.08
- (1|33): Dims=666x40 Mem(GB)=0.03 Int/s=1.94e+06 T(MD)=0.013709 T(Ref)=0.026967 T(Ref/MD)=1.97
- (3|33): Dims=285x40 Mem(GB)=0.03 Int/s=1.07e+06 T(MD)=0.010651 T(Ref)=0.032501 T(Ref/MD)=3.05
- (1|44): Dims=666x24 Mem(GB)=0.03 Int/s=9.77e+05 T(MD)=0.016368 T(Ref)=0.041960 T(Ref/MD)=2.56
- (4|44): Dims=222x24 Mem(GB)=0.03 Int/s=3.56e+05 T(MD)=0.014959 T(Ref)=0.080278 T(Ref/MD)=5.37
- (1|55): Dims=666x16 Mem(GB)=0.03 Int/s=4.88e+05 T(MD)=0.021851 T(Ref)=0.061096 T(Ref/MD)=2.80
- (5|55): Dims=181x16 Mem(GB)=0.03 Int/s=1.19e+05 T(MD)=0.024355 T(Ref)=0.158376 T(Ref/MD)=6.50
- (1|66): Dims=666x11 Mem(GB)=0.03 Int/s=1.88e+05 T(MD)=0.038937 T(Ref)=0.109421 T(Ref/MD)=2.81
- (6|66): Dims=153x11 Mem(GB)=0.03 Int/s=5.38e+04 T(MD)=0.031260 T(Ref)=0.257584 T(Ref/MD)=8.24

K: [1,10]

results:

- (0|00): Dims=2000x2000 Mem(GB)=0.03 Int/s=2.84e+07 T(MD)=0.140811 T(Ref)=0.559419 T(Ref/MD)=3.97
- (1|00): Dims=666x2000 Mem(GB)=0.03 Int/s=2.46e+07 T(MD)=0.054172 T(Ref)=0.333448 T(Ref/MD)=6.16
- (2|00): Dims=400x2000 Mem(GB)=0.03 Int/s=1.93e+07 T(MD)=0.041505 T(Ref)=0.223142 T(Ref/MD)=5.38
- (4|00): Dims=222x2000 Mem(GB)=0.03 Int/s=1.12e+07 T(MD)=0.039719 T(Ref)=0.199810 T(Ref/MD)=5.03
- (6|00): Dims=153x2000 Mem(GB)=0.03 Int/s=5.78e+06 T(MD)=0.052910 T(Ref)=0.210682 T(Ref/MD)=3.98
- (0|11): Dims=2000x222 Mem(GB)=0.03 Int/s=8.34e+06 T(MD)=0.053226 T(Ref)=0.123572 T(Ref/MD)=2.32
- (1|11): Dims=666x222 Mem(GB)=0.03 Int/s=4.33e+06 T(MD)=0.034117 T(Ref)=0.055394 T(Ref/MD)=1.62
- (2|11): Dims=400x222 Mem(GB)=0.03 Int/s=2.75e+06 T(MD)=0.032271 T(Ref)=0.043515 T(Ref/MD)=1.35
- (4|11): Dims=222x222 Mem(GB)=0.03 Int/s=1.57e+06 T(MD)=0.031442 T(Ref)=0.085160 T(Ref/MD)=2.71
- (6|11): Dims=153x222 Mem(GB)=0.03 Int/s=9.09e+05 T(MD)=0.037347 T(Ref)=0.143546 T(Ref/MD)=3.84
- (1|22): Dims=666x80 Mem(GB)=0.03 Int/s=7.56e+05 T(MD)=0.070463 T(Ref)=0.073324 T(Ref/MD)=1.04
- (2|22): Dims=400x80 Mem(GB)=0.03 Int/s=5.01e+05 T(MD)=0.063823 T(Ref)=0.071738 T(Ref/MD)=1.12
- (1|33): Dims=666x40 Mem(GB)=0.03 Int/s=2.04e+05 T(MD)=0.130304 T(Ref)=0.088675 T(Ref/MD)=0.68
- (3|33): Dims=285x40 Mem(GB)=0.03 Int/s=1.10e+05 T(MD)=0.103407 T(Ref)=0.109609 T(Ref/MD)=1.06
- (1|44): Dims=666x24 Mem(GB)=0.03 Int/s=1.00e+05 T(MD)=0.159279 T(Ref)=0.122080 T(Ref/MD)=0.77
- (4|44): Dims=222x24 Mem(GB)=0.03 Int/s=3.98e+04 T(MD)=0.133999 T(Ref)=0.316153 T(Ref/MD)=2.36
- (1|55): Dims=666x16 Mem(GB)=0.03 Int/s=5.04e+04 T(MD)=0.211577 T(Ref)=0.168167 T(Ref/MD)=0.79
- (5|55): Dims=181x16 Mem(GB)=0.03 Int/s=1.26e+04 T(MD)=0.229068 T(Ref)=0.582615 T(Ref/MD)=2.54
- (1|66): Dims=666x11 Mem(GB)=0.03 Int/s=1.91e+04 T(MD)=0.382718 T(Ref)=0.241306 T(Ref/MD)=0.63
- (6|66): Dims=153x11 Mem(GB)=0.03 Int/s=5.51e+03 T(MD)=0.305579 T(Ref)=0.940390 T(Ref/MD)=3.08

K: [5,10]

results:

- (0|00): Dims=2000x2000 Mem(GB)=0.03 Int/s=5.27e+06 T(MD)=0.758590 T(Ref)=2.549988 T(Ref/MD)=3.36
- (1|00): Dims=666x2000 Mem(GB)=0.03 Int/s=4.89e+06 T(MD)=0.272297 T(Ref)=1.485886 T(Ref/MD)=5.46
- (2|00): Dims=400x2000 Mem(GB)=0.03 Int/s=3.92e+06 T(MD)=0.204051 T(Ref)=0.967341 T(Ref/MD)=4.74
- (4|00): Dims=222x2000 Mem(GB)=0.03 Int/s=2.36e+06 T(MD)=0.188065 T(Ref)=0.843106 T(Ref/MD)=4.48
- (6|00): Dims=153x2000 Mem(GB)=0.03 Int/s=1.22e+06 T(MD)=0.251740 T(Ref)=0.896976 T(Ref/MD)=3.56
- (0|11): Dims=2000x222 Mem(GB)=0.03 Int/s=1.77e+06 T(MD)=0.251495 T(Ref)=0.525504 T(Ref/MD)=2.09
- (1|11): Dims=666x222 Mem(GB)=0.03 Int/s=9.15e+05 T(MD)=0.161653 T(Ref)=0.225574 T(Ref/MD)=1.40
- (2|11): Dims=400x222 Mem(GB)=0.03 Int/s=5.63e+05 T(MD)=0.157677 T(Ref)=0.176721 T(Ref/MD)=1.12
- (4|11): Dims=222x222 Mem(GB)=0.03 Int/s=3.17e+05 T(MD)=0.155286 T(Ref)=0.373190 T(Ref/MD)=2.40
- (6|11): Dims=153x222 Mem(GB)=0.03 Int/s=1.80e+05 T(MD)=0.188998 T(Ref)=0.660673 T(Ref/MD)=3.50
- (1|22): Dims=666x80 Mem(GB)=0.03 Int/s=4.10e+05 T(MD)=0.129915 T(Ref)=0.309504 T(Ref/MD)=2.38
- (2|22): Dims=400x80 Mem(GB)=0.03 Int/s=2.90e+05 T(MD)=0.110239 T(Ref)=0.305731 T(Ref/MD)=2.77
- (1|33): Dims=666x40 Mem(GB)=0.03 Int/s=1.43e+05 T(MD)=0.185913 T(Ref)=0.372687 T(Ref/MD)=2.00
- (3|33): Dims=285x40 Mem(GB)=0.03 Int/s=7.82e+04 T(MD)=0.145776 T(Ref)=0.452813 T(Ref/MD)=3.11
- (1|44): Dims=666x24 Mem(GB)=0.03 Int/s=7.16e+04 T(MD)=0.223169 T(Ref)=0.493475 T(Ref/MD)=2.21
- (4|44): Dims=222x24 Mem(GB)=0.03 Int/s=3.06e+04 T(MD)=0.174301 T(Ref)=1.363822 T(Ref/MD)=7.82
- (1|55): Dims=666x16 Mem(GB)=0.03 Int/s=3.73e+04 T(MD)=0.285685 T(Ref)=0.640736 T(Ref/MD)=2.24
- (5|55): Dims=181x16 Mem(GB)=0.03 Int/s=1.07e+04 T(MD)=0.269810 T(Ref)=2.447256 T(Ref/MD)=9.07
- (1|66): Dims=666x11 Mem(GB)=0.03 Int/s=1.75e+04 T(MD)=0.418852 T(Ref)=0.816134 T(Ref/MD)=1.95
- (6|66): Dims=153x11 Mem(GB)=0.03 Int/s=4.86e+03 T(MD)=0.346573 T(Ref)=3.908241 T(Ref/MD)=11.28

---

md4/avx0

4-center performance test

simd: OFF

cxx: 12.3.0

blas: Intel MKL 2020.4

```
K: [1,1]
results:
- (00|00): Dims=4000x4000 Int/s=1.29e+08 T(MD)=0.124278 T(Ref)=0.567378 T(Ref/MD)=4.57
- (10|00): Dims=1333x4000 Int/s=6.18e+07 T(MD)=0.086221 T(Ref)=0.921887 T(Ref/MD)=10.69
- (11|00): Dims=444x4000 Int/s=3.49e+07 T(MD)=0.050924 T(Ref)=0.352786 T(Ref/MD)=6.93
- (11|11): Dims=444x444 Int/s=5.00e+06 T(MD)=0.039447 T(Ref)=0.091041 T(Ref/MD)=2.31
- (22|00): Dims=160x4000 Int/s=1.18e+07 T(MD)=0.054219 T(Ref)=0.194312 T(Ref/MD)=3.58
- (22|11): Dims=160x444 Int/s=1.17e+06 T(MD)=0.060655 T(Ref)=0.136234 T(Ref/MD)=2.25
- (22|22): Dims=160x160 Int/s=3.89e+05 T(MD)=0.065808 T(Ref)=0.197488 T(Ref/MD)=3.00
- (33|00): Dims=81x4000 Int/s=3.52e+06 T(MD)=0.091997 T(Ref)=0.182973 T(Ref/MD)=1.99
- (33|11): Dims=81x444 Int/s=3.82e+05 T(MD)=0.094063 T(Ref)=0.205590 T(Ref/MD)=2.19
- (33|33): Dims=81x81 Int/s=4.21e+04 T(MD)=0.155700 T(Ref)=0.568572 T(Ref/MD)=3.65
- (44|00): Dims=49x4000 Int/s=1.25e+06 T(MD)=0.156532 T(Ref)=0.342312 T(Ref/MD)=2.19
- (44|11): Dims=49x444 Int/s=1.36e+05 T(MD)=0.160021 T(Ref)=0.325387 T(Ref/MD)=2.03
- (44|44): Dims=49x49 Int/s=5.37e+03 T(MD)=0.446750 T(Ref)=1.568054 T(Ref/MD)=3.51
- (55|00): Dims=33x4000 Int/s=5.04e+05 T(MD)=0.262022 T(Ref)=0.506396 T(Ref/MD)=1.93
- (55|11): Dims=33x444 Int/s=5.67e+04 T(MD)=0.258315 T(Ref)=0.506588 T(Ref/MD)=1.96
- (55|55): Dims=33x33 Int/s=1.41e+03 T(MD)=0.772652 T(Ref)=4.243514 T(Ref/MD)=5.49
- (66|00): Dims=23x4000 Int/s=2.36e+05 T(MD)=0.389310 T(Ref)=0.801214 T(Ref/MD)=2.06
- (66|11): Dims=23x444 Int/s=2.38e+04 T(MD)=0.429250 T(Ref)=0.729898 T(Ref/MD)=1.70
- (66|66): Dims=23x23 Int/s=4.45e+02 T(MD)=1.188273 T(Ref)=17.072680 T(Ref/MD)=14.37
---
K: [1,10]
results:
- (00|00): Dims=4000x4000 Int/s=1.73e+07 T(MD)=0.927237 T(Ref)=3.918484 T(Ref/MD)=4.23
- (10|00): Dims=1333x4000 Int/s=1.02e+07 T(MD)=0.520330 T(Ref)=2.853058 T(Ref/MD)=5.48
- (11|00): Dims=444x4000 Int/s=6.13e+06 T(MD)=0.289636 T(Ref)=1.074018 T(Ref/MD)=3.71
- (11|11): Dims=444x444 Int/s=8.39e+05 T(MD)=0.234888 T(Ref)=0.305777 T(Ref/MD)=1.30
- (22|00): Dims=160x4000 Int/s=2.72e+06 T(MD)=0.235464 T(Ref)=0.577568 T(Ref/MD)=2.45
- (22|11): Dims=160x444 Int/s=2.47e+05 T(MD)=0.287373 T(Ref)=0.437009 T(Ref/MD)=1.52
- (22|22): Dims=160x160 Int/s=8.28e+04 T(MD)=0.309275 T(Ref)=0.754311 T(Ref/MD)=2.44
- (33|00): Dims=81x4000 Int/s=9.46e+05 T(MD)=0.342653 T(Ref)=0.575247 T(Ref/MD)=1.68
- (33|11): Dims=81x444 Int/s=9.15e+04 T(MD)=0.392944 T(Ref)=0.624324 T(Ref/MD)=1.59
```

- (33|33): Dims=81x81 Int/s=7.20e+03 T(MD)=0.911557 T(Ref)=2.116857 T(Ref/MD)=2.32
- (44|00): Dims=49x4000 Int/s=3.93e+05 T(MD)=0.498318 T(Ref)=1.212951 T(Ref/MD)=2.43
- (44|11): Dims=49x444 Int/s=3.45e+04 T(MD)=0.630324 T(Ref)=0.971272 T(Ref/MD)=1.54
- (44|44): Dims=49x49 Int/s=8.30e+02 T(MD)=2.894042 T(Ref)=5.366165 T(Ref/MD)=1.85
- (55|00): Dims=33x4000 Int/s=1.91e+05 T(MD)=0.689907 T(Ref)=1.701663 T(Ref/MD)=2.47
- (55|11): Dims=33x444 Int/s=2.98e+04 T(MD)=0.491111 T(Ref)=1.375642 T(Ref/MD)=2.80
- (55|55): Dims=33x33 Int/s=2.13e+02 T(MD)=5.116598 T(Ref)=11.007244 T(Ref/MD)=2.15
- (66|00): Dims=23x4000 Int/s=9.54e+04 T(MD)=0.964222 T(Ref)=2.342336 T(Ref/MD)=2.43
- (66|11): Dims=23x444 Int/s=9.80e+03 T(MD)=1.041681 T(Ref)=1.793375 T(Ref/MD)=1.72
- (66|66): Dims=23x23 Int/s=6.67e+01 T(MD)=7.934431 T(Ref)=26.630214 T(Ref/MD)=3.36

K: [5,10]

results:

- (00|00): Dims=4000x4000 Int/s=3.34e+06 T(MD)=4.789803 T(Ref)=18.770433 T(Ref/MD)=3.92
- (10|00): Dims=1333x4000 Int/s=2.04e+06 T(MD)=2.615868 T(Ref)=11.181054 T(Ref/MD)=4.27
- (11|00): Dims=444x4000 Int/s=1.21e+06 T(MD)=1.466843 T(Ref)=4.117294 T(Ref/MD)=2.81
- (11|11): Dims=444x444 Int/s=1.67e+05 T(MD)=1.183207 T(Ref)=1.216841 T(Ref/MD)=1.03
- (22|00): Dims=160x4000 Int/s=5.20e+05 T(MD)=1.229596 T(Ref)=2.236302 T(Ref/MD)=1.82
- (22|11): Dims=160x444 Int/s=4.97e+04 T(MD)=1.428273 T(Ref)=1.747221 T(Ref/MD)=1.22
- (22|22): Dims=160x160 Int/s=1.67e+04 T(MD)=1.528659 T(Ref)=3.191304 T(Ref/MD)=2.09
- (33|00): Dims=81x4000 Int/s=1.86e+05 T(MD)=1.738878 T(Ref)=2.279606 T(Ref/MD)=1.31
- (33|11): Dims=81x444 Int/s=1.80e+04 T(MD)=1.993881 T(Ref)=2.465150 T(Ref/MD)=1.24
- (33|33): Dims=81x81 Int/s=1.43e+03 T(MD)=4.588884 T(Ref)=8.847264 T(Ref/MD)=1.93
- (44|00): Dims=49x4000 Int/s=7.93e+04 T(MD)=2.472080 T(Ref)=5.000060 T(Ref/MD)=2.02
- (44|11): Dims=49x444 Int/s=6.94e+03 T(MD)=3.135373 T(Ref)=3.759221 T(Ref/MD)=1.20
- (44|44): Dims=49x49 Int/s=1.68e+02 T(MD)=14.274971 T(Ref)=21.806854 T(Ref/MD)=1.53
- (55|00): Dims=33x4000 Int/s=3.84e+04 T(MD)=3.439534 T(Ref)=6.775555 T(Ref/MD)=1.97
- (55|11): Dims=33x444 Int/s=6.03e+03 T(MD)=2.427981 T(Ref)=5.098447 T(Ref/MD)=2.10
- (55|55): Dims=33x33 Int/s=4.38e+01 T(MD)=24.844422 T(Ref)=39.599114 T(Ref/MD)=1.59
- (66|00): Dims=23x4000 Int/s=1.91e+04 T(MD)=4.822905 T(Ref)=9.082917 T(Ref/MD)=1.88
- (66|11): Dims=23x444 Int/s=1.96e+03 T(MD)=5.218048 T(Ref)=6.450088 T(Ref/MD)=1.24
- (66|66): Dims=23x23 Int/s=1.36e+01 T(MD)=38.878913 T(Ref)=69.891202 T(Ref/MD)=1.80

md4/avx256

```
# 4-center performance test
simd: AVX 256-bits
cxx: Ubuntu Clang 15.0.7
blas: Intel MKL 2020
---
K: [1,1]
results:
- (00|00): Dims=4000x4000 Int/s=1.74e+08 T(MD)=0.091904 T(Ref)=0.676195 T(Ref/MD)=7.36
- (10|00): Dims=1333x4000 Int/s=1.30e+08 T(MD)=0.040862 T(Ref)=1.063305 T(Ref/MD)=26.02
- (11|00): Dims=444x4000 Int/s=6.98e+07 T(MD)=0.025461 T(Ref)=0.418617 T(Ref/MD)=16.44
- (11|11): Dims=444x444 Int/s=1.16e+07 T(MD)=0.016940 T(Ref)=0.102899 T(Ref/MD)=6.07
- (22|00): Dims=160x4000 Int/s=1.75e+07 T(MD)=0.036607 T(Ref)=0.218728 T(Ref/MD)=5.98
- (22|11): Dims=160x444 Int/s=2.16e+06 T(MD)=0.032927 T(Ref)=0.138247 T(Ref/MD)=4.20
- (22|22): Dims=160x160 Int/s=4.85e+05 T(MD)=0.052777 T(Ref)=0.201142 T(Ref/MD)=3.81
- (33|00): Dims=81x4000 Int/s=4.74e+06 T(MD)=0.068414 T(Ref)=0.200472 T(Ref/MD)=2.93
- (33|11): Dims=81x444 Int/s=5.50e+05 T(MD)=0.065330 T(Ref)=0.202598 T(Ref/MD)=3.10
- (33|33): Dims=81x81 Int/s=4.48e+04 T(MD)=0.146305 T(Ref)=0.623309 T(Ref/MD)=4.26
- (44|00): Dims=49x4000 Int/s=1.36e+06 T(MD)=0.143852 T(Ref)=0.389199 T(Ref/MD)=2.71
- (44|11): Dims=49x444 Int/s=1.56e+05 T(MD)=0.139177 T(Ref)=0.333520 T(Ref/MD)=2.40
- (44|44): Dims=49x49 Int/s=6.17e+03 T(MD)=0.389196 T(Ref)=1.685454 T(Ref/MD)=4.33
- (55|00): Dims=33x4000 Int/s=3.86e+05 T(MD)=0.342165 T(Ref)=0.541537 T(Ref/MD)=1.58
- (55|11): Dims=33x444 Int/s=4.13e+04 T(MD)=0.354498 T(Ref)=0.495843 T(Ref/MD)=1.40
- (55|55): Dims=33x33 Int/s=1.30e+03 T(MD)=0.836569 T(Ref)=4.521360 T(Ref/MD)=5.40
- (66|00): Dims=23x4000 Int/s=1.95e+05 T(MD)=0.472261 T(Ref)=0.901858 T(Ref/MD)=1.91
- (66|11): Dims=23x444 Int/s=2.06e+04 T(MD)=0.495053 T(Ref)=0.731796 T(Ref/MD)=1.48
- (66|66): Dims=23x23 Int/s=4.17e+02 T(MD)=1.268576 T(Ref)=16.544293 T(Ref/MD)=13.04
---
```

```
K: [1,10]
results:
- (00|00): Dims=4000x4000 Int/s=2.12e+07 T(MD)=0.754416 T(Ref)=4.088949 T(Ref/MD)=5.42
- (10|00): Dims=1333x4000 Int/s=1.67e+07 T(MD)=0.319014 T(Ref)=3.023223 T(Ref/MD)=9.48
- (11|00): Dims=444x4000 Int/s=1.22e+07 T(MD)=0.145080 T(Ref)=1.135630 T(Ref/MD)=7.83
- (11|11): Dims=444x444 Int/s=2.79e+06 T(MD)=0.070754 T(Ref)=0.324241 T(Ref/MD)=4.58
- (22|00): Dims=160x4000 Int/s=5.10e+06 T(MD)=0.125370 T(Ref)=0.611524 T(Ref/MD)=4.88
- (22|11): Dims=160x444 Int/s=7.69e+05 T(MD)=0.092384 T(Ref)=0.435439 T(Ref/MD)=4.71
- (22|22): Dims=160x160 Int/s=1.06e+05 T(MD)=0.242533 T(Ref)=0.750389 T(Ref/MD)=3.09
- (33|00): Dims=81x4000 Int/s=1.84e+06 T(MD)=0.176529 T(Ref)=0.581308 T(Ref/MD)=3.29
- (33|11): Dims=81x444 Int/s=2.45e+05 T(MD)=0.146520 T(Ref)=0.613366 T(Ref/MD)=4.19
- (33|33): Dims=81x81 Int/s=8.32e+03 T(MD)=0.788267 T(Ref)=2.119756 T(Ref/MD)=2.69
- (44|00): Dims=49x4000 Int/s=7.52e+05 T(MD)=0.260516 T(Ref)=1.238540 T(Ref/MD)=4.75
- (44|11): Dims=49x444 Int/s=9.00e+04 T(MD)=0.241744 T(Ref)=0.960273 T(Ref/MD)=3.97
- (44|44): Dims=49x49 Int/s=1.16e+03 T(MD)=2.067700 T(Ref)=5.544368 T(Ref/MD)=2.68
- (55|00): Dims=33x4000 Int/s=2.75e+05 T(MD)=0.480662 T(Ref)=1.699057 T(Ref/MD)=3.53
- (55|11): Dims=33x444 Int/s=3.07e+04 T(MD)=0.477254 T(Ref)=1.345313 T(Ref/MD)=2.82
- (55|55): Dims=33x33 Int/s=2.54e+02 T(MD)=4.285807 T(Ref)=11.047290 T(Ref/MD)=2.58
- (66|00): Dims=23x4000 Int/s=1.46e+05 T(MD)=0.629630 T(Ref)=2.468934 T(Ref/MD)=3.92
- (66|11): Dims=23x444 Int/s=1.36e+04 T(MD)=0.748490 T(Ref)=1.747204 T(Ref/MD)=2.33
- (66|66): Dims=23x23 Int/s=7.66e+01 T(MD)=6.906924 T(Ref)=26.240454 T(Ref/MD)=3.80
---
K: [5,10]
results:
- (00|00): Dims=4000x4000 Int/s=4.19e+06 T(MD)=3.815355 T(Ref)=20.470300 T(Ref/MD)=5.37
- (10|00): Dims=1333x4000 Int/s=3.32e+06 T(MD)=1.607088 T(Ref)=11.673527 T(Ref/MD)=7.26
- (11|00): Dims=444x4000 Int/s=2.35e+06 T(MD)=0.756506 T(Ref)=4.358971 T(Ref/MD)=5.76
- (11|11): Dims=444x444 Int/s=5.43e+05 T(MD)=0.363201 T(Ref)=1.279528 T(Ref/MD)=3.52
- (22|00): Dims=160x4000 Int/s=9.85e+05 T(MD)=0.649743 T(Ref)=2.355538 T(Ref/MD)=3.63
- (22|11): Dims=160x444 Int/s=1.53e+05 T(MD)=0.465227 T(Ref)=1.764245 T(Ref/MD)=3.79
- (22|22): Dims=160x160 Int/s=2.15e+04 T(MD)=1.189072 T(Ref)=3.159497 T(Ref/MD)=2.66
- (33|00): Dims=81x4000 Int/s=3.55e+05 T(MD)=0.913129 T(Ref)=2.280840 T(Ref/MD)=2.50
- (33|11): Dims=81x444 Int/s=4.88e+04 T(MD)=0.737516 T(Ref)=2.408495 T(Ref/MD)=3.27
```

- (33|33): Dims=81x81 Int/s=1.69e+03 T(MD)=3.883295 T(Ref)=8.833758 T(Ref/MD)=2.27

- (44|00): Dims=49x4000 Int/s=1.45e+05 T(MD)=1.349795 T(Ref)=5.018109 T(Ref/MD)=3.72

- (44|11): Dims=49x444 Int/s=1.75e+04 T(MD)=1.242063 T(Ref)=3.761308 T(Ref/MD)=3.03

- (44|44): Dims=49x49 Int/s=2.37e+02 T(MD)=10.133736 T(Ref)=22.391132 T(Ref/MD)=2.21

- (55|00): Dims=33x4000 Int/s=5.34e+04 T(MD)=2.472587 T(Ref)=6.860127 T(Ref/MD)=2.77

- (55|11): Dims=33x444 Int/s=6.02e+03 T(MD)=2.434146 T(Ref)=5.075524 T(Ref/MD)=2.09

- (55|55): Dims=33x33 Int/s=5.25e+01 T(MD)=20.760107 T(Ref)=39.763740 T(Ref/MD)=1.92

- (66|00): Dims=23x4000 Int/s=2.89e+04 T(MD)=3.182898 T(Ref)=9.352319 T(Ref/MD)=2.94

- (66|11): Dims=23x444 Int/s=2.70e+03 T(MD)=3.778065 T(Ref)=6.293583 T(Ref/MD)=1.67

- (66|66): Dims=23x23 Int/s=1.58e+01 T(MD)=33.441569 T(Ref)=65.419609 T(Ref/MD)=1.96

---

md4/avx512

4-center performance test

simd: AVX512 512-bits

cxx: 14.2.0

blas: Intel MKL 2025

K: [1,1]

results:

- (00|00): Dims=4000x4000 Int/s=2.04e+08 T(MD)=0.078268 T(Ref)=0.694256 T(Ref/MD)=8.87

- (10|00): Dims=1333x4000 Int/s=1.45e+08 T(MD)=0.036848 T(Ref)=0.963175 T(Ref/MD)=26.14

- (11|00): Dims=444x4000 Int/s=8.52e+07 T(MD)=0.020854 T(Ref)=0.367725 T(Ref/MD)=17.63

- (11|11): Dims=444x444 Int/s=1.23e+07 T(MD)=0.016088 T(Ref)=0.092679 T(Ref/MD)=5.76

- (22|00): Dims=160x4000 Int/s=1.99e+07 T(MD)=0.032241 T(Ref)=0.209146 T(Ref/MD)=6.49

- (22|11): Dims=160x444 Int/s=2.95e+06 T(MD)=0.024109 T(Ref)=0.150900 T(Ref/MD)=6.26

- (22|22): Dims=160x160 Int/s=7.11e+05 T(MD)=0.036005 T(Ref)=0.217999 T(Ref/MD)=6.05

- (33|00): Dims=81x4000 Int/s=5.85e+06 T(MD)=0.055393 T(Ref)=0.195808 T(Ref/MD)=3.53

- (33|11): Dims=81x444 Int/s=6.71e+05 T(MD)=0.053599 T(Ref)=0.216385 T(Ref/MD)=4.04

- (33|33): Dims=81x81 Int/s=5.87e+04 T(MD)=0.111690 T(Ref)=0.645833 T(Ref/MD)=5.78

```
- (44|00): Dims=49x4000 Int/s=1.52e+06 T(MD)=0.129315 T(Ref)=0.391137 T(Ref/MD)=3.02
- (44|11): Dims=49x444 Int/s=1.73e+05 T(MD)=0.125497 T(Ref)=0.348988 T(Ref/MD)=2.78
- (44|44): Dims=49x49 Int/s=8.19e+03 T(MD)=0.293341 T(Ref)=2.089201 T(Ref/MD)=7.12
- (55|00): Dims=33x4000 Int/s=4.12e+05 T(MD)=0.320460 T(Ref)=0.564778 T(Ref/MD)=1.76
- (55|11): Dims=33x444 Int/s=4.75e+04 T(MD)=0.308288 T(Ref)=0.529526 T(Ref/MD)=1.72
- (55|55): Dims=33x33 Int/s=1.70e+03 T(MD)=0.642135 T(Ref)=5.675152 T(Ref/MD)=8.84
- (66|00): Dims=23x4000 Int/s=2.18e+05 T(MD)=0.421474 T(Ref)=0.795681 T(Ref/MD)=1.89
- (66|11): Dims=23x444 Int/s=2.54e+04 T(MD)=0.402651 T(Ref)=0.813876 T(Ref/MD)=2.02
- (66|66): Dims=23x23 Int/s=5.57e+02 T(MD)=0.949866 T(Ref)=19.836773 T(Ref/MD)=20.88
---
K: [1,10]
results:
- (00|00): Dims=4000x4000 Int/s=2.10e+07 T(MD)=0.760931 T(Ref)=4.442230 T(Ref/MD)=5.84
- (10|00): Dims=1333x4000 Int/s=1.71e+07 T(MD)=0.312643 T(Ref)=3.150621 T(Ref/MD)=10.08
- (11|00): Dims=444x4000 Int/s=1.35e+07 T(MD)=0.131449 T(Ref)=1.204767 T(Ref/MD)=9.17
- (11|11): Dims=444x444 Int/s=3.14e+06 T(MD)=0.062822 T(Ref)=0.328549 T(Ref/MD)=5.23
- (22|00): Dims=160x4000 Int/s=4.76e+06 T(MD)=0.134438 T(Ref)=0.656726 T(Ref/MD)=4.88
- (22|11): Dims=160x444 Int/s=1.05e+06 T(MD)=0.067890 T(Ref)=0.487897 T(Ref/MD)=7.19
- (22|22): Dims=160x160 Int/s=1.54e+05 T(MD)=0.166343 T(Ref)=0.832335 T(Ref/MD)=5.00
- (33|00): Dims=81x4000 Int/s=2.11e+06 T(MD)=0.153366 T(Ref)=0.634841 T(Ref/MD)=4.14
- (33|11): Dims=81x444 Int/s=3.14e+05 T(MD)=0.114655 T(Ref)=0.675328 T(Ref/MD)=5.89
- (33|33): Dims=81x81 Int/s=1.21e+04 T(MD)=0.542041 T(Ref)=1.991579 T(Ref/MD)=3.67
- (44|00): Dims=49x4000 Int/s=7.83e+05 T(MD)=0.250477 T(Ref)=1.379460 T(Ref/MD)=5.51
- (44|11): Dims=49x444 Int/s=1.02e+05 T(MD)=0.212514 T(Ref)=1.061394 T(Ref/MD)=4.99
- (44|44): Dims=49x49 Int/s=1.51e+03 T(MD)=1.585211 T(Ref)=6.142423 T(Ref/MD)=3.87
- (55|00): Dims=33x4000 Int/s=2.86e+05 T(MD)=0.461062 T(Ref)=1.890966 T(Ref/MD)=4.10
- (55|11): Dims=33x444 Int/s=3.27e+04 T(MD)=0.447955 T(Ref)=1.465383 T(Ref/MD)=3.27
- (55|55): Dims=33x33 Int/s=3.22e+02 T(MD)=3.378628 T(Ref)=13.541241 T(Ref/MD)=4.01
- (66|00): Dims=23x4000 Int/s=1.49e+05 T(MD)=0.616854 T(Ref)=2.548128 T(Ref/MD)=4.13
- (66|11): Dims=23x444 Int/s=1.61e+04 T(MD)=0.634490 T(Ref)=1.993622 T(Ref/MD)=3.14
- (66|66): Dims=23x23 Int/s=1.02e+02 T(MD)=5.194551 T(Ref)=31.890377 T(Ref/MD)=6.14
---
K: [5,10]
```

results:

- (00|00): Dims=4000x4000 Int/s=4.05e+06 T(MD)=3.947860 T(Ref)=21.914376 T(Ref/MD)=5.55
- (10|00): Dims=1333x4000 Int/s=3.28e+06 T(MD)=1.625323 T(Ref)=12.626055 T(Ref/MD)=7.77
- (11|00): Dims=444x4000 Int/s=2.61e+06 T(MD)=0.680771 T(Ref)=4.893885 T(Ref/MD)=7.19
- (11|11): Dims=444x444 Int/s=6.38e+05 T(MD)=0.309139 T(Ref)=1.424701 T(Ref/MD)=4.61
- (22|00): Dims=160x4000 Int/s=9.32e+05 T(MD)=0.686369 T(Ref)=2.650609 T(Ref/MD)=3.86
- (22|11): Dims=160x444 Int/s=2.10e+05 T(MD)=0.337551 T(Ref)=1.991444 T(Ref/MD)=5.90
- (22|22): Dims=160x160 Int/s=3.13e+04 T(MD)=0.816727 T(Ref)=3.556554 T(Ref/MD)=4.35
- (33|00): Dims=81x4000 Int/s=4.02e+05 T(MD)=0.804999 T(Ref)=2.579133 T(Ref/MD)=3.20
- (33|11): Dims=81x444 Int/s=6.41e+04 T(MD)=0.560666 T(Ref)=2.712962 T(Ref/MD)=4.84
- (33|33): Dims=81x81 Int/s=2.45e+03 T(MD)=2.677037 T(Ref)=8.015649 T(Ref/MD)=2.99
- (44|00): Dims=49x4000 Int/s=1.51e+05 T(MD)=1.298886 T(Ref)=5.749362 T(Ref/MD)=4.43
- (44|11): Dims=49x444 Int/s=2.01e+04 T(MD)=1.079886 T(Ref)=4.215290 T(Ref/MD)=3.90
- (44|44): Dims=49x49 Int/s=3.08e+02 T(MD)=7.798052 T(Ref)=24.423005 T(Ref/MD)=3.13
- (55|00): Dims=33x4000 Int/s=5.14e+04 T(MD)=2.565968 T(Ref)=7.785246 T(Ref/MD)=3.03
- (55|11): Dims=33x444 Int/s=5.95e+03 T(MD)=2.463406 T(Ref)=5.646781 T(Ref/MD)=2.29
- (55|55): Dims=33x33 Int/s=6.64e+01 T(MD)=16.390127 T(Ref)=48.745797 T(Ref/MD)=2.97
- (66|00): Dims=23x4000 Int/s=2.64e+04 T(MD)=3.482804 T(Ref)=10.327861 T(Ref/MD)=2.97
- (66|11): Dims=23x444 Int/s=2.86e+03 T(MD)=3.564869 T(Ref)=7.166851 T(Ref/MD)=2.01
- (66|66): Dims=23x23 Int/s=2.12e+01 T(MD)=25.006015 T(Ref)=85.688896 T(Ref/MD)=3.43

---

md4/neon

4-center performance test

simd: NEON 128-bits

cxx: Homebrew Clang 17.0.6

K: [1,1]

results:

- (00|00): Dims=4000x4000 Int/s=2.23e+08 T(MD)=0.071904 T(Ref)=0.376965 T(Ref/MD)=5.24

```
- (10|00): Dims=1333x4000 Int/s=1.40e+08 T(MD)=0.038072 T(Ref)=0.447865 T(Ref/MD)=11.76
- (11|00): Dims=444x4000 Int/s=6.46e+07 T(MD)=0.027486 T(Ref)=0.187330 T(Ref/MD)=6.82
- (11|11): Dims=444x444 Int/s=9.92e+06 T(MD)=0.019881 T(Ref)=0.052460 T(Ref/MD)=2.64
- (22|00): Dims=160x4000 Int/s=1.64e+07 T(MD)=0.038958 T(Ref)=0.124426 T(Ref/MD)=3.19
- (22|11): Dims=160x444 Int/s=2.02e+06 T(MD)=0.035118 T(Ref)=0.095928 T(Ref/MD)=2.73
- (22|22): Dims=160x160 Int/s=2.83e+05 T(MD)=0.090460 T(Ref)=0.138464 T(Ref/MD)=1.53
- (33|00): Dims=81x4000 Int/s=4.69e+06 T(MD)=0.069072 T(Ref)=0.102495 T(Ref/MD)=1.48
- (33|11): Dims=81x444 Int/s=5.71e+05 T(MD)=0.062969 T(Ref)=0.131886 T(Ref/MD)=2.09
- (33|33): Dims=81x81 Int/s=4.73e+04 T(MD)=0.138836 T(Ref)=0.438886 T(Ref/MD)=3.16
- (44|00): Dims=49x4000 Int/s=1.65e+06 T(MD)=0.119079 T(Ref)=0.236671 T(Ref/MD)=1.99
- (44|11): Dims=49x444 Int/s=1.98e+05 T(MD)=0.109731 T(Ref)=0.206745 T(Ref/MD)=1.88
- (44|44): Dims=49x49 Int/s=1.12e+04 T(MD)=0.213634 T(Ref)=0.969638 T(Ref/MD)=4.54
- (55|00): Dims=33x4000 Int/s=5.27e+05 T(MD)=0.250386 T(Ref)=0.345710 T(Ref/MD)=1.38
- (55|11): Dims=33x444 Int/s=5.80e+04 T(MD)=0.252510 T(Ref)=0.333770 T(Ref/MD)=1.32
- (55|55): Dims=33x33 Int/s=3.02e+03 T(MD)=0.361132 T(Ref)=2.091964 T(Ref/MD)=5.79
- (66|00): Dims=23x4000 Int/s=2.24e+05 T(MD)=0.411605 T(Ref)=0.572960 T(Ref/MD)=1.39
- (66|11): Dims=23x444 Int/s=2.41e+04 T(MD)=0.422948 T(Ref)=0.504664 T(Ref/MD)=1.19
- (66|66): Dims=23x23 Int/s=8.31e+02 T(MD)=0.636768 T(Ref)=5.845459 T(Ref/MD)=9.18
---
K: [1,10]
results:
- (00|00): Dims=4000x4000 Int/s=2.75e+07 T(MD)=0.582726 T(Ref)=2.313563 T(Ref/MD)=3.97
- (10|00): Dims=1333x4000 Int/s=1.79e+07 T(MD)=0.297768 T(Ref)=1.602305 T(Ref/MD)=5.38
- (11|00): Dims=444x4000 Int/s=1.27e+07 T(MD)=0.140002 T(Ref)=0.601546 T(Ref/MD)=4.30
- (11|11): Dims=444x444 Int/s=2.17e+06 T(MD)=0.090725 T(Ref)=0.172803 T(Ref/MD)=1.90
- (22|00): Dims=160x4000 Int/s=4.34e+06 T(MD)=0.147532 T(Ref)=0.361462 T(Ref/MD)=2.45
- (22|11): Dims=160x444 Int/s=6.60e+05 T(MD)=0.107704 T(Ref)=0.328917 T(Ref/MD)=3.05
- (22|22): Dims=160x160 Int/s=6.06e+04 T(MD)=0.422313 T(Ref)=0.532573 T(Ref/MD)=1.26
- (33|00): Dims=81x4000 Int/s=1.59e+06 T(MD)=0.203630 T(Ref)=0.345840 T(Ref/MD)=1.70
- (33|11): Dims=81x444 Int/s=2.36e+05 T(MD)=0.152239 T(Ref)=0.439307 T(Ref/MD)=2.89
- (33|33): Dims=81x81 Int/s=1.77e+04 T(MD)=0.371628 T(Ref)=2.032377 T(Ref/MD)=5.47
- (44|00): Dims=49x4000 Int/s=7.08e+05 T(MD)=0.276723 T(Ref)=0.921879 T(Ref/MD)=3.33
- (44|11): Dims=49x444 Int/s=9.94e+04 T(MD)=0.218978 T(Ref)=0.649600 T(Ref/MD)=2.97
```

```
- (44|44): Dims=49x49 Int/s=2.66e+03 T(MD)=0.903954 T(Ref)=3.796203 T(Ref/MD)=4.20
- (55|00): Dims=33x4000 Int/s=2.95e+05 T(MD)=0.447549 T(Ref)=1.256315 T(Ref/MD)=2.81
- (55|11): Dims=33x444 Int/s=2.95e+04 T(MD)=0.496292 T(Ref)=0.882629 T(Ref/MD)=1.78
- (55|55): Dims=33x33 Int/s=7.98e+02 T(MD)=1.365095 T(Ref)=6.214692 T(Ref/MD)=4.55
- (66|00): Dims=23x4000 Int/s=1.46e+05 T(MD)=0.628688 T(Ref)=1.741580 T(Ref/MD)=2.77
- (66|11): Dims=23x444 Int/s=1.15e+04 T(MD)=0.890000 T(Ref)=1.148722 T(Ref/MD)=1.29
- (66|66): Dims=23x23 Int/s=2.68e+02 T(MD)=1.973954 T(Ref)=11.432848 T(Ref/MD)=5.79
---
K: [5,10]
results:
- (00|00): Dims=4000x4000 Int/s=5.19e+06 T(MD)=3.080646 T(Ref)=10.885649 T(Ref/MD)=3.53
- (10|00): Dims=1333x4000 Int/s=3.50e+06 T(MD)=1.524558 T(Ref)=6.918902 T(Ref/MD)=4.54
- (11|00): Dims=444x4000 Int/s=2.40e+06 T(MD)=0.741145 T(Ref)=2.416382 T(Ref/MD)=3.26
- (11|11): Dims=444x444 Int/s=4.31e+05 T(MD)=0.457505 T(Ref)=0.707883 T(Ref/MD)=1.55
- (22|00): Dims=160x4000 Int/s=8.51e+05 T(MD)=0.752439 T(Ref)=1.410875 T(Ref/MD)=1.88
- (22|11): Dims=160x444 Int/s=1.31e+05 T(MD)=0.544287 T(Ref)=1.352350 T(Ref/MD)=2.48
- (22|22): Dims=160x160 Int/s=1.22e+04 T(MD)=2.094669 T(Ref)=2.284036 T(Ref/MD)=1.09
- (33|00): Dims=81x4000 Int/s=2.88e+05 T(MD)=1.123325 T(Ref)=1.475045 T(Ref/MD)=1.31
- (33|11): Dims=81x444 Int/s=4.45e+04 T(MD)=0.807317 T(Ref)=1.847502 T(Ref/MD)=2.29
- (33|33): Dims=81x81 Int/s=3.53e+03 T(MD)=1.857510 T(Ref)=9.291979 T(Ref/MD)=5.00
- (44|00): Dims=49x4000 Int/s=1.38e+05 T(MD)=1.423663 T(Ref)=3.942204 T(Ref/MD)=2.77
- (44|11): Dims=49x444 Int/s=1.95e+04 T(MD)=1.113556 T(Ref)=2.605054 T(Ref/MD)=2.34
- (44|44): Dims=49x49 Int/s=5.39e+02 T(MD)=4.455403 T(Ref)=16.265384 T(Ref/MD)=3.65
- (55|00): Dims=33x4000 Int/s=5.77e+04 T(MD)=2.288717 T(Ref)=5.310534 T(Ref/MD)=2.32
- (55|11): Dims=33x444 Int/s=5.74e+03 T(MD)=2.554241 T(Ref)=3.340269 T(Ref/MD)=1.31
- (55|55): Dims=33x33 Int/s=1.63e+02 T(MD)=6.669330 T(Ref)=24.556899 T(Ref/MD)=3.68
- (66|00): Dims=23x4000 Int/s=2.85e+04 T(MD)=3.227072 T(Ref)=6.893355 T(Ref/MD)=2.14
- (66|11): Dims=23x444 Int/s=2.24e+03 T(MD)=4.560403 T(Ref)=3.986003 T(Ref/MD)=0.87
- (66|66): Dims=23x23 Int/s=5.54e+01 T(MD)=9.542024 T(Ref)=35.952034 T(Ref/MD)=3.77
```

md4/simint.avx256

```
# 4-center performance test
simd: AVX 256-bits
cxx: Ubuntu Clang 15.0.7
num_threads: 1
---
K: [1,1]
results:
- (00|00): Dims=1000x1000 Int/s=1.68e+08 T(MD)=0.005939 T(Ref)=0.010588 T(Ref/MD)=1.78
- (10|00): Dims=333x1000 Int/s=1.17e+08 T(MD)=0.002839 T(Ref)=0.005466 T(Ref/MD)=1.92
- (11|00): Dims=111x1000 Int/s=6.26e+07 T(MD)=0.001774 T(Ref)=0.002903 T(Ref/MD)=1.64
- (11|11): Dims=111x111 Int/s=1.04e+07 T(MD)=0.001182 T(Ref)=0.001466 T(Ref/MD)=1.24
- (22|00): Dims=40x1000 Int/s=1.58e+07 T(MD)=0.002534 T(Ref)=0.002910 T(Ref/MD)=1.15
- (22|11): Dims=40x111 Int/s=1.92e+06 T(MD)=0.002308 T(Ref)=0.002819 T(Ref/MD)=1.22
- (22|22): Dims=40x40 Int/s=4.45e+05 T(MD)=0.003597 T(Ref)=0.007024 T(Ref/MD)=1.95
- (33|00): Dims=20x1000 Int/s=4.42e+06 T(MD)=0.004530 T(Ref)=0.007936 T(Ref/MD)=1.75
- (33|11): Dims=20x111 Int/s=5.14e+05 T(MD)=0.004322 T(Ref)=0.008733 T(Ref/MD)=2.02
- (33|33): Dims=20x20 Int/s=4.81e+04 T(MD)=0.008323 T(Ref)=0.027713 T(Ref/MD)=3.33
- (44|00): Dims=12x1000 Int/s=1.42e+06 T(MD)=0.008467 T(Ref)=0.013526 T(Ref/MD)=1.60
- (44|11): Dims=12x111 Int/s=1.62e+05 T(MD)=0.008202 T(Ref)=0.018239 T(Ref/MD)=2.22
- (44|44): Dims=12x12 Int/s=6.39e+03 T(MD)=0.022521 T(Ref)=0.069707 T(Ref/MD)=3.10
- (55|00): Dims=8x1000 Int/s=4.30e+05 T(MD)=0.018620 T(Ref)=0.086876 T(Ref/MD)=4.67
- (55|11): Dims=8x111 Int/s=4.55e+04 T(MD)=0.019502 T(Ref)=0.045179 T(Ref/MD)=2.32
- (55|55): Dims=8x8 Int/s=1.40e+03 T(MD)=0.045594 T(Ref)=0.226102 T(Ref/MD)=4.96
---
K: [1,10]
results:
- (00|00): Dims=1000x1000 Int/s=2.01e+07 T(MD)=0.049830 T(Ref)=0.085374 T(Ref/MD)=1.71
- (10|00): Dims=333x1000 Int/s=1.57e+07 T(MD)=0.021163 T(Ref)=0.046509 T(Ref/MD)=2.20
- (11|00): Dims=111x1000 Int/s=1.14e+07 T(MD)=0.009763 T(Ref)=0.023494 T(Ref/MD)=2.41
- (11|11): Dims=111x111 Int/s=2.63e+06 T(MD)=0.004679 T(Ref)=0.009443 T(Ref/MD)=2.02
- (22|00): Dims=40x1000 Int/s=4.75e+06 T(MD)=0.008420 T(Ref)=0.015375 T(Ref/MD)=1.83
```

```
- (22|11): Dims=40x111 Int/s=7.00e+05 T(MD)=0.006347 T(Ref)=0.012632 T(Ref/MD)=1.99
- (22|22): Dims=40x40 Int/s=1.00e+05 T(MD)=0.015984 T(Ref)=0.036275 T(Ref/MD)=2.27
- (33|00): Dims=20x1000 Int/s=1.79e+06 T(MD)=0.011198 T(Ref)=0.021981 T(Ref/MD)=1.96
- (33|11): Dims=20x111 Int/s=2.39e+05 T(MD)=0.009286 T(Ref)=0.034440 T(Ref/MD)=3.71
- (33|33): Dims=20x20 Int/s=8.19e+03 T(MD)=0.048820 T(Ref)=0.132611 T(Ref/MD)=2.72
- (44|00): Dims=12x1000 Int/s=7.61e+05 T(MD)=0.015759 T(Ref)=0.034404 T(Ref/MD)=2.18
- (44|11): Dims=12x111 Int/s=9.18e+04 T(MD)=0.014505 T(Ref)=0.072801 T(Ref/MD)=5.02
- (44|44): Dims=12x12 Int/s=1.18e+03 T(MD)=0.122024 T(Ref)=0.270427 T(Ref/MD)=2.22
- (55|00): Dims=8x1000 Int/s=2.95e+05 T(MD)=0.027136 T(Ref)=0.120548 T(Ref/MD)=4.44
- (55|11): Dims=8x111 Int/s=3.31e+04 T(MD)=0.026796 T(Ref)=0.121234 T(Ref/MD)=4.52
- (55|55): Dims=8x8 Int/s=2.69e+02 T(MD)=0.238328 T(Ref)=0.548395 T(Ref/MD)=2.30
---
K: [5,10]
results:
- (00|00): Dims=1000x1000 Int/s=3.94e+06 T(MD)=0.253572 T(Ref)=0.385691 T(Ref/MD)=1.52
- (10|00): Dims=333x1000 Int/s=3.07e+06 T(MD)=0.108500 T(Ref)=0.201475 T(Ref/MD)=1.86
- (11|00): Dims=111x1000 Int/s=2.16e+06 T(MD)=0.051281 T(Ref)=0.102895 T(Ref/MD)=2.01
- (11|11): Dims=111x111 Int/s=5.17e+05 T(MD)=0.023809 T(Ref)=0.046761 T(Ref/MD)=1.96
- (22|00): Dims=40x1000 Int/s=9.30e+05 T(MD)=0.042997 T(Ref)=0.076245 T(Ref/MD)=1.77
- (22|11): Dims=40x111 Int/s=1.45e+05 T(MD)=0.030676 T(Ref)=0.056967 T(Ref/MD)=1.86
- (22|22): Dims=40x40 Int/s=2.03e+04 T(MD)=0.078815 T(Ref)=0.162994 T(Ref/MD)=2.07
- (33|00): Dims=20x1000 Int/s=3.50e+05 T(MD)=0.057152 T(Ref)=0.086883 T(Ref/MD)=1.52
- (33|11): Dims=20x111 Int/s=4.77e+04 T(MD)=0.046564 T(Ref)=0.148498 T(Ref/MD)=3.19
- (33|33): Dims=20x20 Int/s=1.65e+03 T(MD)=0.241960 T(Ref)=0.604580 T(Ref/MD)=2.50
- (44|00): Dims=12x1000 Int/s=1.51e+05 T(MD)=0.079696 T(Ref)=0.131148 T(Ref/MD)=1.65
- (44|11): Dims=12x111 Int/s=1.86e+04 T(MD)=0.071805 T(Ref)=0.318143 T(Ref/MD)=4.43
- (44|44): Dims=12x12 Int/s=2.36e+02 T(MD)=0.610365 T(Ref)=1.161744 T(Ref/MD)=1.90
- (55|00): Dims=8x1000 Int/s=5.58e+04 T(MD)=0.143368 T(Ref)=0.273131 T(Ref/MD)=1.91
- (55|11): Dims=8x111 Int/s=6.36e+03 T(MD)=0.139603 T(Ref)=0.461961 T(Ref/MD)=3.31
- (55|55): Dims=8x8 Int/s=5.35e+01 T(MD)=1.195378 T(Ref)=1.973968 T(Ref/MD)=1.65
```

md4/thread

```
# 4-center performance test
simd: AVX 256-bits
cxx: Ubuntu Clang 15.0.7
---
K: [1,1]
results:
- (00|00): Dims=4000x4000 Int/s=5.25e+08 T(MD)=0.030471
- (11|11): Dims=444x444 Int/s=4.34e+07 T(MD)=0.004544
- (22|22): Dims=160x160 Int/s=1.96e+06 T(MD)=0.013094
- (33|33): Dims=81x81 Int/s=1.98e+05 T(MD)=0.033116
- (44|44): Dims=49x49 Int/s=2.43e+04 T(MD)=0.098749
- (55|55): Dims=33x33 Int/s=3.52e+03 T(MD)=0.309327

# 4-center performance test
simd: OFF
cxx: Ubuntu Clang 15.0.7
---
K: [1,1]
results:
- (00|00): Dims=4000x4000 Int/s=2.33e+08 T(MD)=0.068549
- (11|11): Dims=444x444 Int/s=7.83e+06 T(MD)=0.025164
- (22|22): Dims=160x160 Int/s=8.36e+05 T(MD)=0.030623
- (33|33): Dims=81x81 Int/s=1.11e+05 T(MD)=0.059336
- (44|44): Dims=49x49 Int/s=1.60e+04 T(MD)=0.149661
- (55|55): Dims=33x33 Int/s=5.53e+03 T(MD)=0.197020
```

md4/threads

```
# 4-center performance test
simd: OFF
cxx: 12.3.0
num_threads: 6
---
K: [1,1]
results:
- (00|00): Dims=4000x4000 Int/s=6.98e+08 T(MD)=0.022935
- (10|00): Dims=1333x4000 Int/s=3.21e+08 T(MD)=0.016620
- (11|11): Dims=444x444 Int/s=2.72e+07 T(MD)=0.007260
- (22|22): Dims=160x160 Int/s=1.96e+06 T(MD)=0.013065
- (33|33): Dims=81x81 Int/s=2.06e+05 T(MD)=0.031836
- (44|44): Dims=49x49 Int/s=2.60e+04 T(MD)=0.092334
- (55|55): Dims=33x33 Int/s=6.42e+03 T(MD)=0.169675
- (66|66): Dims=23x23 Int/s=1.50e+03 T(MD)=0.352530
---
K: [1,10]
results:
- (00|00): Dims=4000x4000 Int/s=8.79e+07 T(MD)=0.182006
- (10|00): Dims=1333x4000 Int/s=5.04e+07 T(MD)=0.105762
- (11|11): Dims=444x444 Int/s=4.23e+06 T(MD)=0.046629
- (22|22): Dims=160x160 Int/s=4.02e+05 T(MD)=0.063605
- (33|33): Dims=81x81 Int/s=3.31e+04 T(MD)=0.198044
- (44|44): Dims=49x49 Int/s=3.78e+03 T(MD)=0.635451
- (55|55): Dims=33x33 Int/s=9.18e+02 T(MD)=1.186449
- (66|66): Dims=23x23 Int/s=2.55e+02 T(MD)=2.076590
---
K: [5,10]
results:
- (00|00): Dims=4000x4000 Int/s=1.52e+07 T(MD)=1.055285
- (10|00): Dims=1333x4000 Int/s=8.90e+06 T(MD)=0.599284
```

```
- (11|11): Dims=444x444 Int/s=7.23e+05 T(MD)=0.272732
- (22|22): Dims=160x160 Int/s=6.90e+04 T(MD)=0.370959
- (33|33): Dims=81x81 Int/s=5.79e+03 T(MD)=1.132351
- (44|44): Dims=49x49 Int/s=6.82e+02 T(MD)=3.522004
- (55|55): Dims=33x33 Int/s=1.67e+02 T(MD)=6.506491
- (66|66): Dims=23x23 Int/s=4.71e+01 T(MD)=11.232766

# 4-center performance test
simd: AVX 256-bits
cxx: Ubuntu Clang 15.0.7
num_threads: 6
---
K: [1,1]
results:
- (00|00): Dims=4000x4000 Int/s=5.79e+08 T(MD)=0.027637
- (10|00): Dims=1333x4000 Int/s=4.50e+08 T(MD)=0.011844
- (11|11): Dims=444x444 Int/s=4.37e+07 T(MD)=0.004510
- (22|22): Dims=160x160 Int/s=2.24e+06 T(MD)=0.011415
- (33|33): Dims=81x81 Int/s=2.02e+05 T(MD)=0.032486
- (44|44): Dims=49x49 Int/s=2.33e+04 T(MD)=0.103211
- (55|55): Dims=33x33 Int/s=3.48e+03 T(MD)=0.313320
- (66|66): Dims=23x23 Int/s=1.10e+03 T(MD)=0.481879
---
K: [1,10]
results:
- (00|00): Dims=4000x4000 Int/s=8.42e+07 T(MD)=0.189990
- (10|00): Dims=1333x4000 Int/s=6.93e+07 T(MD)=0.076987
- (11|11): Dims=444x444 Int/s=1.16e+07 T(MD)=0.016928
- (22|22): Dims=160x160 Int/s=4.47e+05 T(MD)=0.057232
- (33|33): Dims=81x81 Int/s=3.30e+04 T(MD)=0.198873
- (44|44): Dims=49x49 Int/s=4.38e+03 T(MD)=0.548563
- (55|55): Dims=33x33 Int/s=6.58e+02 T(MD)=1.655202
- (66|66): Dims=23x23 Int/s=1.97e+02 T(MD)=2.687084
```

```
---

K: [5,10]

results:

- (00|00): Dims=4000x4000 Int/s=1.64e+07 T(MD)=0.976542

- (10|00): Dims=1333x4000 Int/s=1.33e+07 T(MD)=0.402078

- (11|11): Dims=444x444 Int/s=2.12e+06 T(MD)=0.092900

- (22|22): Dims=160x160 Int/s=8.15e+04 T(MD)=0.314287

- (33|33): Dims=81x81 Int/s=6.21e+03 T(MD)=1.056567

- (44|44): Dims=49x49 Int/s=8.53e+02 T(MD)=2.815768

- (55|55): Dims=33x33 Int/s=1.31e+02 T(MD)=8.322948

- (66|66): Dims=23x23 Int/s=3.95e+01 T(MD)=13.387820
```
